# BGSC-Net: Boundary-guided semantic compensation network for remote sensing image segmentation

**DOI:** 10.1371/journal.pone.0345762

**Published:** 2026-03-31

**Authors:** Xin Wang, Zhe Lu, Qun Yang, Jia Lu, Hao Yang, Qin Qin, Guan Lian, Jiawei Wang

**Affiliations:** 1 School of Computer and Information Security, Guilin University of Electronic Technology, Guilin, China; 2 School of Computer Engineering, Guilin University of Electronic Technology, Beihai, China; 3 School of Electronic Information, Guilin University of Electronic Technology, Beihai, China; 4 Guilin Blue Harbor Technology Co., Ltd, Guilin, China; 5 School of Architecture and Transportation Engineering, Guilin University of Electronic Technology, Guilin, China; 6 Dakang Supply Chain (Guangxi) Group Co., Ltd., Nanning, China; Shandong Agricultural University, CHINA

## Abstract

Deep learning has recently made remarkable progress in remote sensing image segmentation, with hybrid architectures that integrate convolutional neural networks (CNNs) and Transformers emerging as a promising solution, particularly for high-resolution imagery. However, challenges remain in complex remote sensing scenes, particularly in capturing detailed boundary structures and small-scale targets. One key limitation lies in the suboptimal cross-level feature fusion within the encoder, resulting in semantic misalignment that hinders the precise segmentation of small objects and fine structural details. Additionally, during the decoding stage, the lack of explicit boundary guidance frequently causes the loss of edge information during feature reconstruction, compromising the delineation of object contours in intricate environments. To address these issues, We propose a novel hybrid architecture named Boundary-Guided Semantic Compensation Network (BGSC-Net). Our framework integrates two key components: a Cross-Level Semantic Compensation Module (CLSCM) that dynamically fuses high-level semantics with low-level spatial details to enhance small object segmentation, and an Auxiliary Boundary Supervision Module (ABSM) that enhances structural modeling for blurry or complex boundaries through explicit boundary modeling and an auxiliary supervision strategy based on joint optimization of the edge and main segmentation branches. Experiments show that BGSC-Net achieves superior segmentation performance, with mIoU scores of 87.57% on Potsdam, 85.61% on Vaihingen, 55.05% on LoveDA, and 74.77% on UAVid. To further validate its generalization capability in specialized fine-grained segmentation tasks, we evaluated the model on our challenging self-constructed Mangrove Species Fine-grained Segmentation Dataset (MSFSD), where it achieved an mIoU of 89.58%, confirming its practical utility for precise mangrove species mapping.

## 1. Introduction

High-resolution urban–rural remote sensing image segmentation, as an interdisciplinary field combining computer vision and geographic information science, aims to achieve pixel-level accurate segmentation and semantic interpretation of multiple land-cover categories such as buildings, roads, vehicles, farmland, vegetation, and water bodies [1]. This technology plays a vital role in various urban applications, including city planning, infrastructure development, land use monitoring, and change detection [2]. In rural contexts, it provides essential technical support for agricultural management, farmland monitoring, water resource planning, and rural revitalization. As such, it has become a key enabler of smart cities, precision agriculture, and sustainable development.

As the resolution of remote sensing imagery continues to improve, the texture and structural complexity of objects in urban–rural scenes have increased significantly [3]. These images often contain rich details, diverse categories, and similar textures, accompanied by background noise and interference, posing greater challenges to segmentation tasks. Deep convolutional neural networks (DCNNs) have recently achieved remarkable success in remote sensing image segmentation [4,5]. Classical models such as FCN [6], UNet [7], and PSPNet [8] have been widely applied to multi-class land-cover segmentation. Nonetheless, the inherent locality of CNNs’ receptive fields limits their capacity to model global contextual information, which is essential for capturing multi-scale spatial distributions and semantic structures in complex remote sensing scenes [9–12]. Transformer-based architectures have gained increasing adoption in remote sensing image segmentation due to their self-attention mechanisms, which effectively capture long-range semantic dependencies [13,14], thus addressing these limitations. Representative models such as Swin Transformer [15] and SegFormer [16] have shown strong capabilities in global context modeling [17]. However, these models still face challenges in fine-grained boundary delineation, often leading to blurred object contours and the omission of small targets, while also introducing considerable computational overhead.HSIs offer rich, comprehensive data due to their high resolution and wide spectral range, but their abundance might make analysis more difficult.

Recently, hybrid architectures that combine CNNs for local feature extraction with Transformers for global context modeling have increasingly been adopted as a leading strategy to address these challenges. For example, Chen et al. [18] proposed TransUNet, the first hybrid semantic segmentation framework that integrates convolutional neural networks (CNNs) with Transformers by employing a Transformer encoder for strong global representation and a CNN decoder to recover spatial details, achieving a balanced tradeoff between global context and local feature refinement. Zhang et al. [19] presented ST-UNet, which employs a parallel dual-encoder architecture combining Swin Transformer and CNN. to fuse contextual semantics with spatial structural features, significantly improving the model’s ability to recognize objects across multiple scales. Wang et al. [10] developed UNetFormer, which utilizes a CNN encoder paired with a Transformer decoder, introducing an efficient attention mechanism in the decoder to effectively capture both long-range dependencies and fine-grained spatial details simultaneously. To further improve contextual modeling, Xin et al. [20] proposed a hybrid architecture, which introduced a geometric prior-guided interactive network (GPINet) that employs dual-branch encoders with local-global interaction modules to bilaterally couple CNN and transformer features for enhanced contextual understanding.

These hybrid models have achieved encouraging results in complex urban–rural remote sensing scenarios, but several key limitations remain. Transformer-based encoders, while effective at modeling global context, often struggle to capture fine-grained structures and local details. Dual-encoder frameworks, on the other hand, tend to introduce redundant features and suffer from inconsistency across multi-scale representations due to suboptimal fusion strategies. Moreover, both types of architectures typically incur high computational costs, limiting their applicability in resource-constrained environments [21]. In view of the shortcomings inherent in the above two types of architectures, we employ a relatively lightweight design that combines a computationally efficient CNN encoder with a Transformer decoder to balance local feature extraction and global context modeling. Nevertheless, this design still has room for improvement: the CNN encoder often fails to capture long-range semantic dependencies, and feature fusion may lead to semantic misalignment. Furthermore, the Transformer decoder tends to lose edge information of remote sensing images during feature reconstruction due to the lack of boundary guidance, which compromises segmentation accuracy for small objects and complex structures [22].

In response to the above-mentioned issues, we propose a boundary-guided semantic compensation network (BGSC-Net) with a hybrid architecture of a CNN-based encoder and a Transformer-based decoder, which consists of two key modules: the cross-level semantic compensation module (CLSCM) and the decoder-driven auxiliary boundary supervision module (ABSM). Specifically, On the encoder side, CLSCM overcomes the limitations of static feature fusion by employing dynamic weighting and multi-level spatial-channel attention, enhancing the recognition of small targets and fine-grained structures. It also enables Transformer-like global context modeling with lower computational cost, thereby effectively enhancing the semantic consistency and spatial integrity of feature representations. On the decoder side, ABSM leverages multi-scale decoder features in the auxiliary boundary branch to enhance structural detail representation while preserving semantic completeness. Through the joint optimization of local detail enhancement, explicit boundary modeling, and auxiliary supervision, it comprehensively strengthens boundary representation. This effectively compensates for the Transformer decoder’s limitations in fine-grained boundary modeling and small-object segmentation, significantly improving boundary completeness, continuity, and class separability. Building on these two modules, BGSC-Net achieves a favorable balance between high-resolution segmentation accuracy and computational efficiency, offering a novel solution for the accurate delineation and boundary modeling of complex small objects in remote sensing images. Overall, our main contributions are as summarized follows:

- We propose a hybrid model named BGSC-Net, which introduces a dynamic semantic compensation mechanism in the CNN encoder to enhance semantic representation, and incorporates an auxiliary branch in the Transformer decoder to guide boundary refinement. This design effectively addresses the limitations of CNN-Transformer hybrid architectures while incurring only a small additional computational cost.
- We propose a cross-level semantic compensation module (CLSCM), which leverages a dynamic semantic compensation mechanism to effectively fuse high-level semantics with low-level spatial details, thereby significantly improving the segmentation of small targets and fine-grained structures.
- We propose a decoder-driven auxiliary boundary supervision module (ABSM), which fully leverages the dual advantages of multi-scale decoder features in semantic representation and structural restoration. By utilizing an auxiliary edge branch to guide the main segmentation branch in boundary refinement.

## 2. Related works

### 2.1. CNN-Transformer hybrid architecture

In recent years, hybrid architectures combining Convolutional Neural Networks (CNNs) and Transformers have been extensively explored for high-resolution remote sensing image segmentation, aiming to effectively balance local detail modeling with global context extraction [14,23]. These hybrid architectures generally fall into three typical paradigms. The first uses a Transformer encoder and CNN decoder (e.g., TransUNet [18]), where self-attention effectively captures global context, and the CNN decoder gradually restores spatial resolution, enabling detailed reconstruction of high-resolution features. The second approach adopts a CNN encoder and Transformer decoder (e.g., BAFormer [24], CMTFNet [25], UNetFormer [10]), with CNNs efficiently extracting fine-grained local features and the Transformer decoder fusing multi-scale semantics while modeling long-range dependencies. This structure maintains both detailed representation and global understanding. The third approach uses dual encoders-CNN and Transformer-to capture local details and global context in parallel or sequentially, with feature fusion in the decoder (e.g., DEDNet [26], ST-UNet [27]). This approach enhances segmentation accuracy by leveraging complementary strengths of both architectures. Despite their successes, these hybrid models still suffer from several notable limitations. First, architectures with Transformer-based encoders are often less effective at capturing local details and fine-grained structures. Second, dual-encoder frameworks tend to introduce redundant representations and feature inconsistencies due to suboptimal fusion strategies. Both architectures also share the drawback of high computational complexity. Lastly, hybrid designs with a CNN encoder and Transformer decoder tend to inadequately capture long-range semantic relationships in the encoder, while the decoder frequently loses fine-grained edge details during feature reconstruction, compromising the accurate segmentation of small objects and complex structures. Recently, a new family of architectures based on state-space models (SSMs), exemplified by the Mamba architecture, has emerged as an efficient alternative for long-range dependency modeling. Unlike self-attention in Transformers, which explicitly computes pairwise interactions with quadratic complexity, Mamba employs state-space modules to model sequential feature state interactions with linear complexity. This enables effective global context modeling while reducing computational burden. UrbanSSF [28] introduces such a Mamba-based decoder framework for very high-resolution (VHR) urban remote sensing image segmentation, leveraging Feature State Interaction (FSI) modules to capture sequential dependencies across multi-scale feature states. This approach facilitates enhanced cross-phase information fusion and improves semantic consistency in complex scenes. Mamba-based models mainly focus on modeling feature sequences and may overlook important spatial details and boundary information. This can make them less effective at capturing fine structures and small objects in very high-resolution images. Therefore, methods that enhance frequency and spatial details are still needed to improve segmentation accuracy.

### 2.2. Cross-layer feature fusion in remote sensing segmentation

In recent years, researchers have proposed numerous improvement strategies focused on cross-level feature fusion, multi-dimensional attention mechanisms, and semantic embedding optimization, aiming to enhance the collaborative representation of low-level spatial details and high-level semantics in remote sensing image segmentation. thereby alleviating semantic misalignment and improving small object detection performance. For instance, Li et al. [29] proposed A2-FPN, which introduces attention-based adaptive feature fusion to enhance semantic consistency across scales and improve small object detection. Nevertheless, its reliance on spatial attention alone may lead to suboptimal feature alignment in complex scenes. Li et al. [30] proposed MAResU-Net, which enhances boundary details and small object perception via multi-scale residual attention. Yet, its reliance on convolutions for compensating low-level spatial details limits the effective integration of high-level semantics, thereby reducing segmentation accuracy in complex scenes. To further improve contextual modeling, Xin et al. [31] proposed SAPNet by analyzing the attention bias problem in existing methods and designing a synergistic attention module (SAM) to jointly model spatial and channel affinities, which significantly enhances contextual perception and achieves superior segmentation performance on multiple benchmarks. However, its coupling strategy still lacks explicit decoupling between low-level spatial details and high-level semantics. Zhang et al. [32] introduced the semantic embedding branch (SEB) in ExFuse to enhance the semantic awareness of low-level features, improving fusion performance. Despite this, its fixed structure lacks adaptability to varying task scenarios. To address the limitations of fixed fusion strategies, Xin et al. [33] proposed DDFNet, a dual-domain decoupled fusion network that selectively fuses high and low-frequency components from spatial and frequency domains via cross-attention. By introducing the DDFF module.and a high-order geometric prior module, DDFNet effectively captures local textures and global semantics, achieving excellent performance on multiple benchmarks. Hwang et al. [11] proposed SFA-Net, featuring a feature adjustment module (FAM) that aligns and reconstructs multi-scale features from the EfficientNet backbone to enhance semantic consistency. Even so, its channel-based fusion limits spatial detail awareness, especially in boundary refinement and small object detection. Liu et al. [34] proposed CM-UNet, incorporating a multi-scale attention aggregation (MSAA) module to fuse features across encoding layers with attention weighting. While effective in representation enhancement, it struggles to decouple low-level spatial details from high-level semantics, leading to redundancy and semantic conflict that impair boundary accuracy. More recently, Xin et al. [35] proposed EAAHNet, the first fully hyperbolic neural network for remote sensing image semantic segmentation, and introduced a Euclidean affinity-augmented hyperbolic attention module to jointly model contextual dependencies. This attention fusion strategy significantly enhances pixel-level semantic recognition, achieving competitive performance on multiple benchmarks. Nevertheless, its complex geometric modeling increases computational overhead and may limit deployment efficiency in large-scale applications.

In summary, while these methods advance feature fusion and attention mechanisms in remote sensing segmentation, they commonly suffer from several drawbacks: reliance on single-dimensional attention leads to suboptimal feature alignment; low-level convolutions hinder high-level semantics integration; fixed fusion structures lack adaptability across diverse scenarios; and insufficient separation between spatial details and semantic representations often causes redundancy and conflicts. These issues restrict the model’s ability to capture fine-grained semantics and detect small objects effectively.

### 2.3. Boundary enhancement in complex remote sensing scenes

In complex remote sensing image segmentation tasks, the integrity, continuity, and class separability of object boundaries are key factors for improving overall segmentation accuracy. However, objects such as buildings, roads, farmland, and forests in remote sensing imagery are often closely intertwined and share similar spectral and texture features, leading to common issues such as blurred or broken boundaries and class confusion, which severely compromise segmentation accuracy.

In response to these issues, current research primarily focuses on two aspects: structural module design and loss function optimization, proposing various boundary enhancement strategies [25,36]. In terms of structural module design, existing methods can be broadly categorized into two types. The first type involves multi-scale boundary enhancement modules, which explicitly extract boundary information using multi-level features and edge detection operators, thereby improving boundary integrity and continuity. Kang et al. [37] introduced a multi-scale deformable attention mechanism and a local adaptive edge detection module (LAMBA) in their DAENet, significantly boosting fine-grained boundary segmentation performance. Additionally, Xin et al. [38] proposed a novel frequency decoupling attention module (FDAM) to separately refine high and low-frequency components of learned representations. The enhanced high-frequency features facilitate precise edge and detail extraction, which is critical for accurate boundary delineation, while the optimized low-frequency components provide robust global structural and contextual understanding, effectively enhancing boundary segmentation in complex scenes. The second type consists of edge-aware attention module, which dynamically focus on boundary regions through spatial or channel attention mechanisms to improve boundary segmentation accuracy and class separability. For example, Liu et al. [39] developed an Edge-aware Semantic Segmentation model that enhances boundary regions using a spatial attention mechanism, thereby improving segmentation performance. These methods have achieved promising results in complex urban–rural remote sensing scenario [25,36,40]. Regarding loss function optimization, researchers have introduced boundary-aware loss functions to strengthen edge perception. For example, MKANet’s Sobel Boundary Loss imposes stronger constraints on edge pixels, effectively improving the segmentation of small objects and fine details [41]. Liu et al. [42] proposed ERN, which enhances boundary awareness through multiple edge supervisions by utilizing low-level features maps and edge labels generated from the ground-truth boundary gradients. By applying edge loss only to the shallower layers of the encoder during training, ERN significantly improves boundary segmentation accuracy.

It can be observed from the aforementioned analysis that despite promising advances in boundary enhancement through structural modules and loss function optimization, existing methods still face several challenges. Structurally, many approaches overly rely on low-level features, resulting in insufficient integration of high-level semantics and leading to limited boundary clarity and continuity [10,11,36,43]. Additionally, computational costs remain high, restricting practical deployment. From the loss function perspective, boundary supervision often depends on encoder features or raw images that lack semantic richness and contain noise, which hampers precise boundary guidance. Although decoder features naturally fuse high-level semantics with structural details and support progressive refinement, they remain underutilized in explicit boundary modeling, which limits the ability to capture fine object boundaries in complex, high-resolution remote sensing scenes.

## 3. Materials and methods

### 3.1. Overview structure

**Fig 1:**
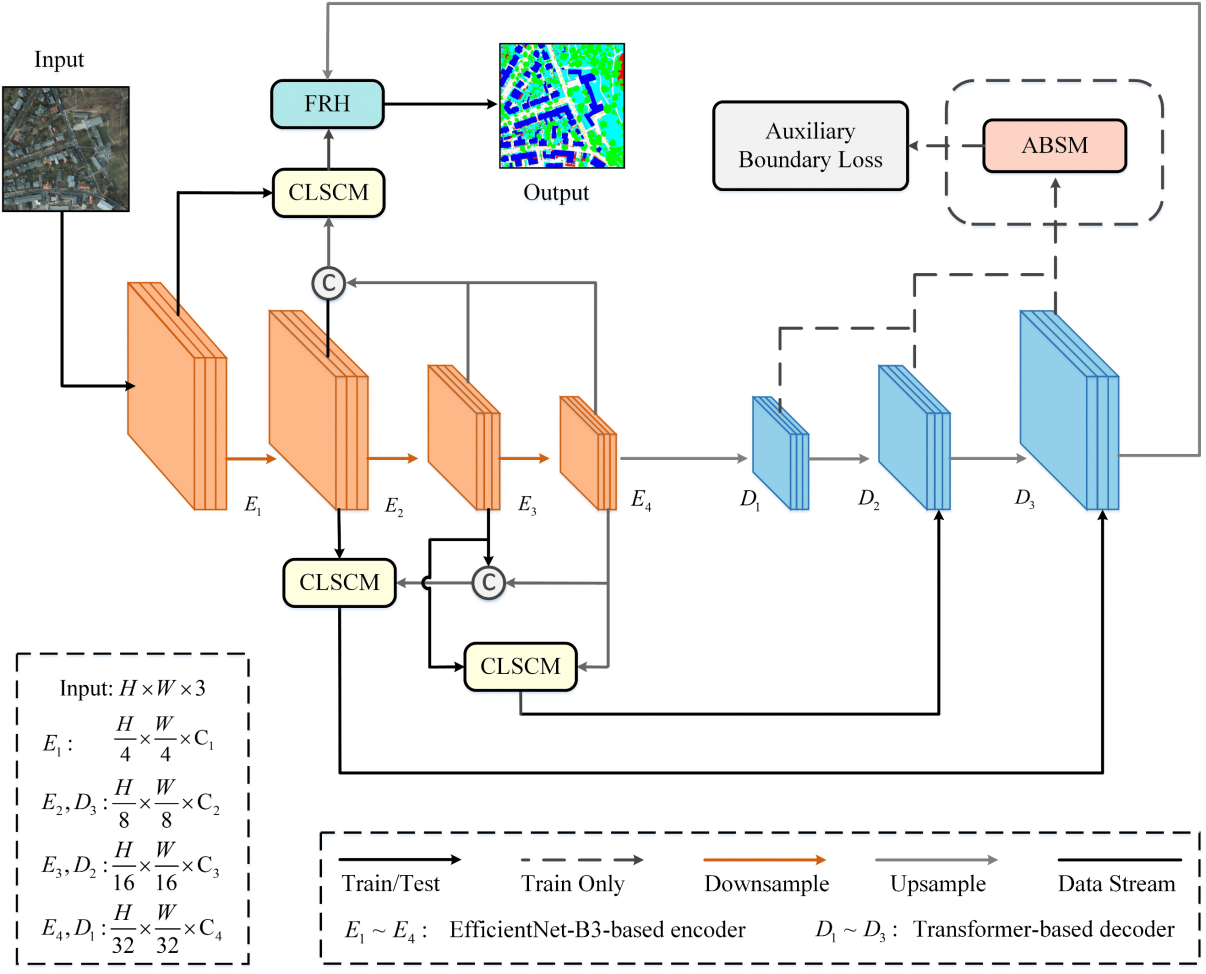
The overall architecture of BGSC-Net.

We propose a novel remote sensing image segmentation network named boundary-guided semantic compensation network (BGSC-Net), as illustrated in Fig 1. The network is built upon the UNetFormer [10] architecture, adopting a hybrid design with a CNN-based encoder and a Transformer-based decoder. To enhance spatial–semantic fusion, the feature refinement head (FRH) from the original model is retained at the end of the network, enabling more effective integration of spatial details and contextual information while refining the final feature representations [10,11].

The core of the proposed BGSC-Net consists of four main components: the EfficientNet-B3-based encoder, the Transformer-based decoder, the cross-level semantic compensation module (CLSCM), and the auxiliary boundary supervision module (ABSM). The encoder efficiently extracts multi-scale local features, while CLSCMs are embedded at encoder stages, $E_{\text{1}}$, $E_{\text{2}}$,and $E_{\text{3}}$ to inject high-level semantics into low-level features. This dynamic semantic compensation enhances the representation of small targets and fine-grained structures [32]. The decoder employs global–local Transformer block to capture long-range dependencies and aggregate contextual information. Meanwhile, ABSM takes decoder features from stages, $D_{\text{1}}$, $D_{\text{2}}$,and $D_{\text{3}}$ as input to construct an auxiliary boundary supervision branch. By introducing auxiliary loss during training, ABSM explicitly guides the network to focus on boundary regions, thereby improving boundary completeness and class separability. In the following subsections, we elaborate on the CNN-Based encoder, Transformer-Based decoder, CLSCM, and ABSM components in detail, with a focus on how each contributes to the overall performance of BGSC-Net.

### 3.2. Efficient CNN-based encoder

**Fig 2:**
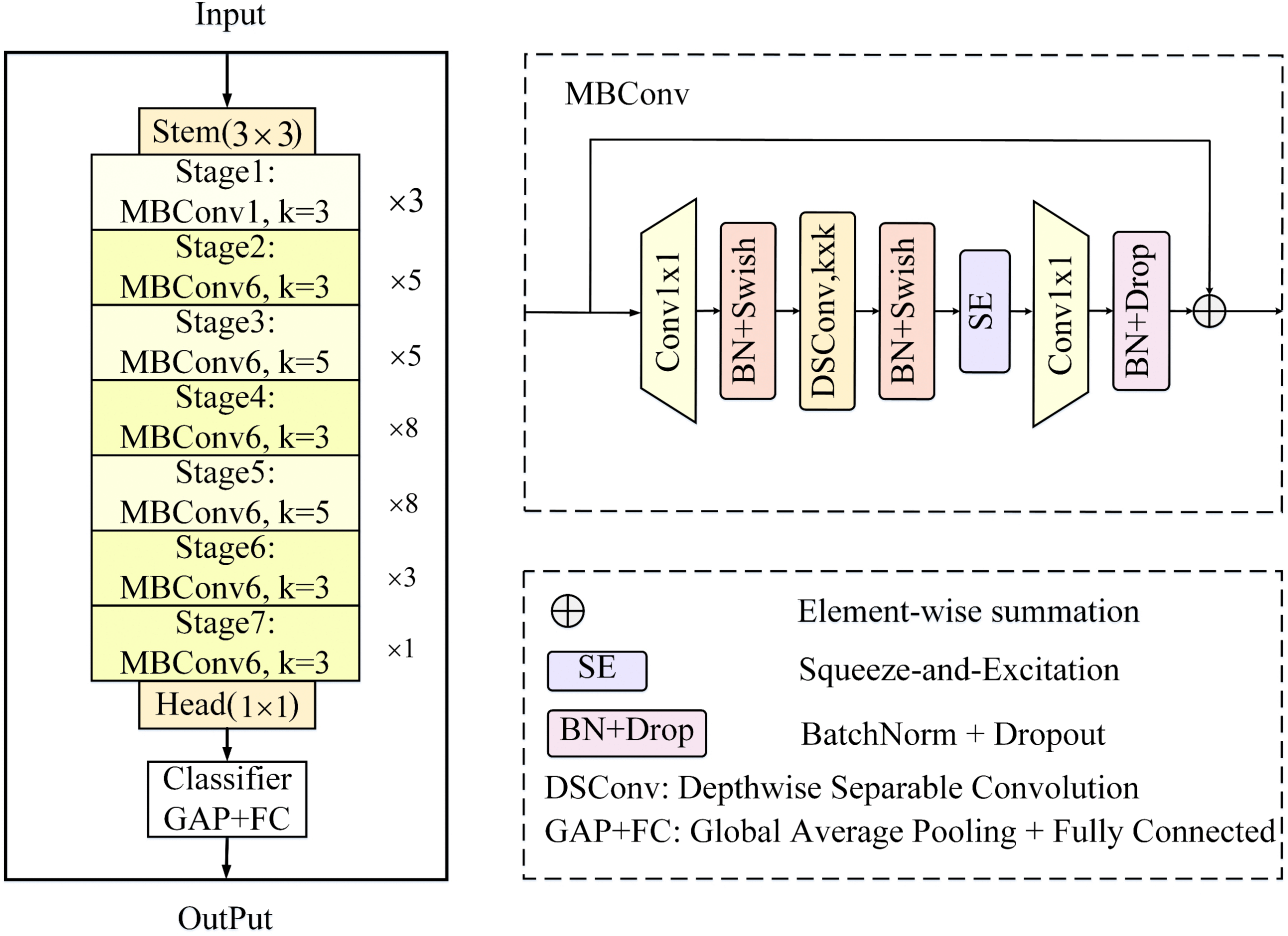
The overall architecture of EfficientNet-B3.


In the proposed BGSC-Net, efficient CNN-based EfficientNet-B3 is used as the encoder backbone (denoted astoin Fig 1). Originally introduced by Tan [44], it employs a compound scaling method that jointly optimizes network depth, width, and input resolution, achieving strong performance with moderate computational cost. As illustrated in Fig 2, the core structure of EfficientNet-B3 consists of seven stages built from stacked MBConv modules, which adopt an inverted residual structure. Each MBConv begins with a 1×1 convolution to expand channels (expansion factor r), followed by a 3×3 or 5×5 depthwise separable convolution for efficient spatial feature extraction. A squeeze-and-excitation (SE) module then recalibrates channel-wise responses to enhance focus on relevant regions. Finally, another 1 × 1 convolution projects the features back to the original dimension, with a residual connection enabling feature reuse and cross-layer information flow [44,45]. The formula is described as follows:

$$MBConv\left(x\right)=SE\left(\;DepthwiseConv\;\left(\sigma \left(Conv_{1\times 1}\left(x\right)\right)\right)\right),$$ (1)

Where $Conv_{1\times 1}(\cdot )$ denotes the expansion layer that multiplies the input channel dimension by the expansion factor; σ(·) represents the activation function (Swish); and SE(·) denotes the squeeze-and-excitation module.

In BGSC-Net, Stages 2 to 5 of EfficientNet-B3 (corresponding to $E_{\text{1}}$ to $E_{\text{4}}$ in Fig 1) are selected as inputs to the encoder. These stages produce feature maps at resolutions of 48, 136, 232, and 384 (denoted as $C_{\text{1}}$ to $C_{\text{4}}$ in Fig 1). This configuration fully leverages the feature extraction capabilities of EfficientNet-B3 in complex remote sensing scenes and provides a strong foundation for high-precision segmentation tasks.

### 3.3. Transformer-based decoder

**Fig 3:**
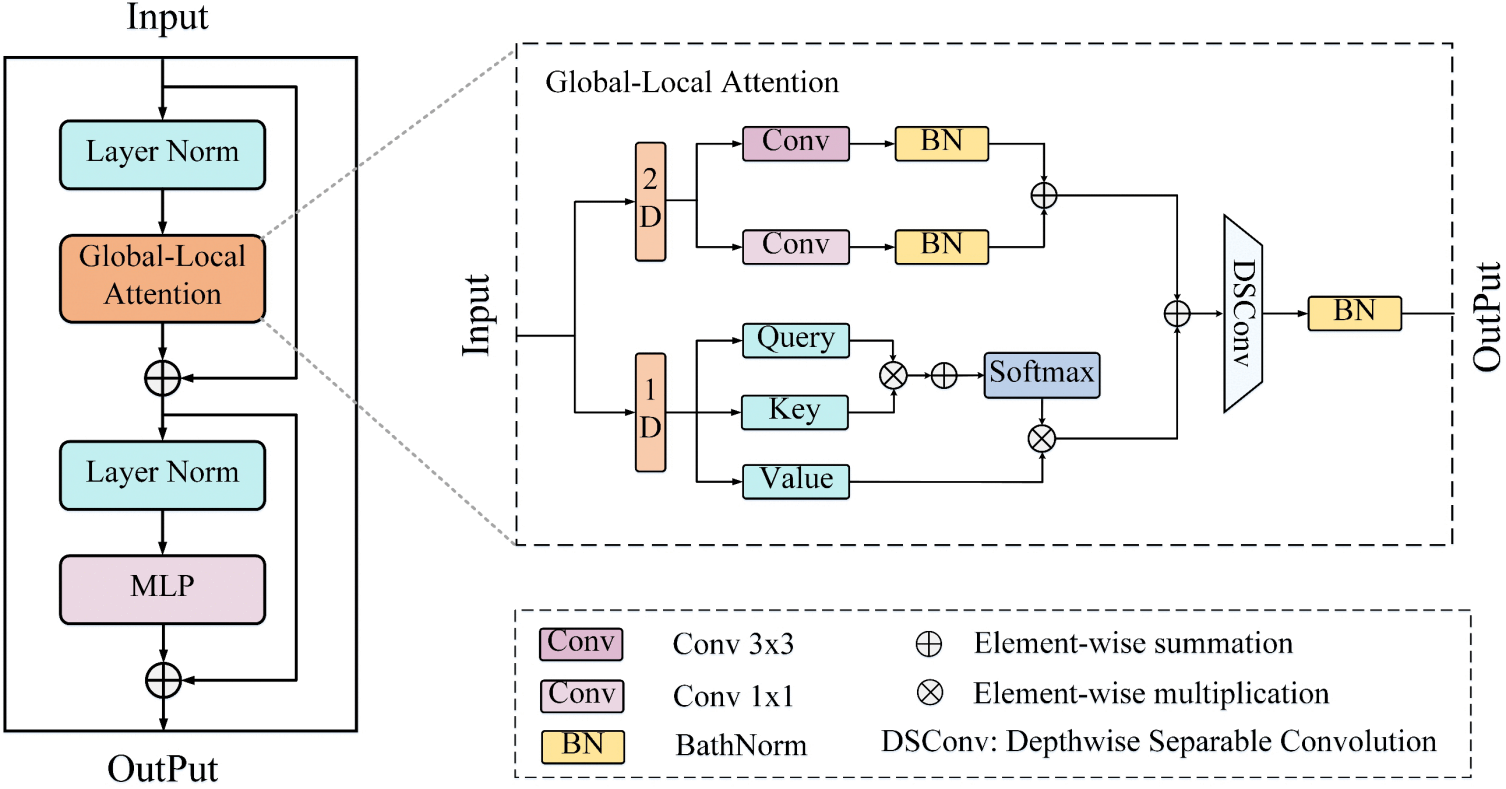
The detailed architecture of the GLTB.

In the proposed BGSC-Net, the Transformer-based decoder (denoted as $D_{\text{1}}$ to $D_{\text{3}}$ in Fig 1) adopts the modular design of UNetFormer [10]. It stacks three global-local Transformer blocks (GLTBs), as illustrated in Fig 3, to progressively recover high-resolution feature representations. Each GLTB consists of a global-local attention, a multilayer perceptron (MLP), and two batch normalization layers, with residual connections between blocks to facilitate efficient feature flow.

The global-local attention module consists of two branches. The global branch uses multi-head self-attention with a window interaction strategy to capture long-range dependencies and reinforces global semantic consistency via horizontal and vertical pooling [11]. The local branch extracts fine-grained spatial details through parallel standard convolutions with kernel sizes 3 and 1, followed by batch normalization to enhance boundary and structural representations. The outputs from both branches are fused via an additional depth-wise separable convolution and batch normalization, producing a unified representation that balances global context with local detail. This design enables the decoder to preserve spatial precision while capturing rich semantics, significantly boosting segmentation performance in complex remote sensing scenes.

### 3.4. Cross-level semantic compensation module (CLSCM)

In semantic segmentation, the fusion of low-level and high-level features enables the model to capture both fine-grained spatial details and abstract semantic information. Existing methods-such as direct concatenation, simple addition, channel weighting, or attention mechanisms like squeeze-and-excitation (SE) and convolutional block attention module (CBAM) have made progress, yet they often suffer from semantic misalignment and inadequate representation of small objects. This is primarily due to their reliance on static or coarsely weighted fusion strategies, which fail to dynamically reconcile the semantic and resolution gaps between different feature levels.

**Fig 4:**
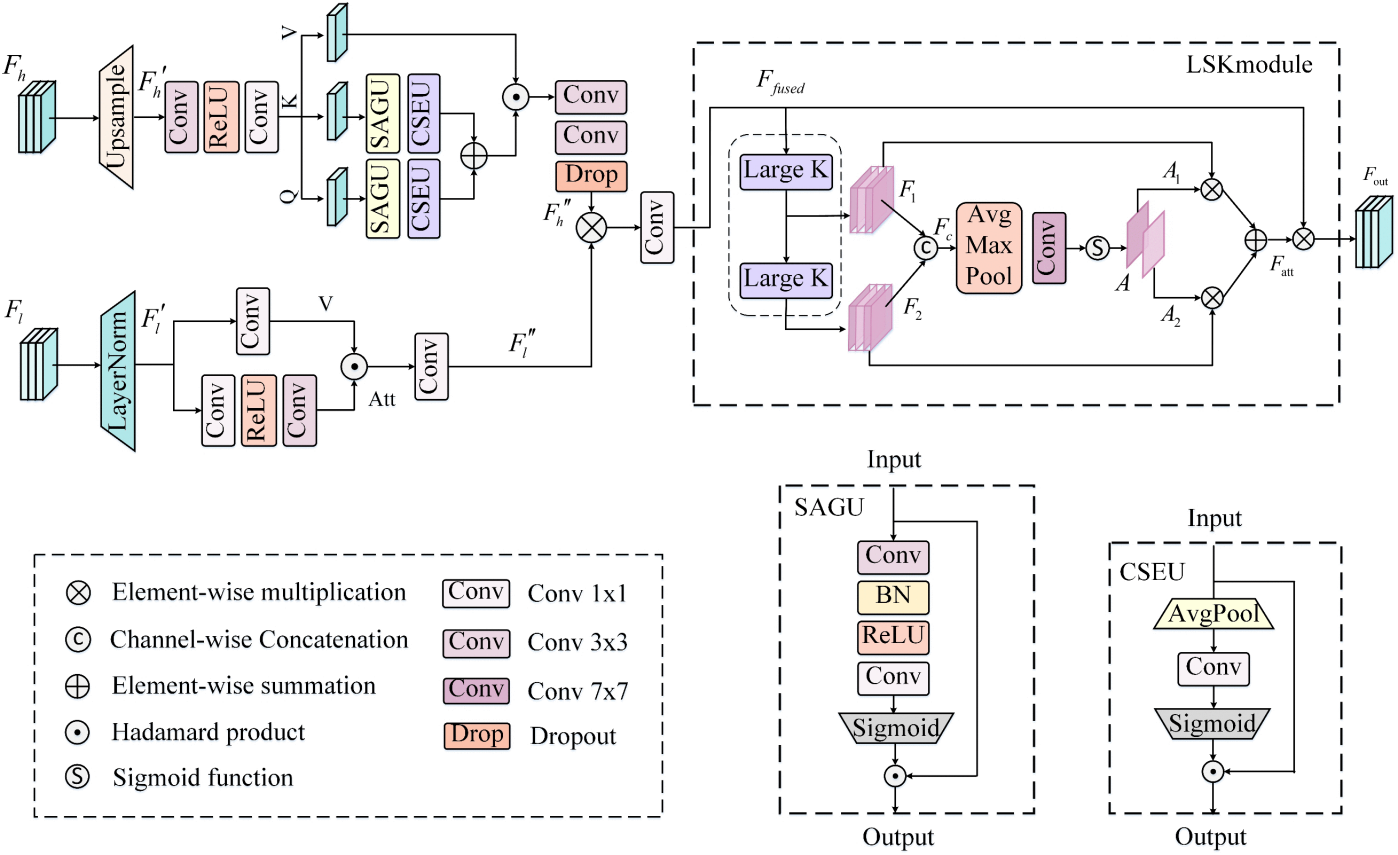
The detailed architecture of the proposed CLSCM.

To address these issues, In the proposed BGSC-Net, we propose a cross-level semantic compensation module (CLSCM), which introduces a dynamic semantic compensation mechanism, Specifically, CLSCM regards high-level semantic features as adaptive guidance signals that dynamically compensate low-level spatial representations, rather than merely fusing them through fixed or symmetric operations.Although CLSCM shares certain structural similarities with existing fusion paradigms such as ExFuse [32] and MAResU-Net [30] (e.g., concatenation and attention-based weighting), its fundamental difference lies in the role of attention.In CLSCM, attention weights are explicitly interpreted as semantic compensation coefficients,which modulate the strength, location, and semantic relevance of cross-level information transfer.This design enables CLSCM to selectively inject high-level semantics into low-level features only where semantic ambiguity or object incompleteness exists, rather than uniformly enhancing all regions. The detailed structure is illustrated in Fig 4.

The workflow of the CLSCM is as follows: first, high-level features from different levels are aggregated and aligned. Specifically, at each encoder stage $E_{i}$ (i =1,2,3), all deeper-layer features (from $E_{i+1}$ to $E_{\text{4}}$) are concatenated along the channel dimension to integrate richer high-level semantic information. The concatenated tensor is then processed using a pixelshuffle upsampling operation, Unlike the bilinear interpolation used in ExFuse, PixelShuffle explicitly redistributes a portion of channel information into the spatial dimension, enabling a channel-to-space mapping that enhances spatial resolution and detail representation while avoiding interpolation-induced smoothing effects. As a result, high-frequency structural information can be better preserved.Moreover, during spatial upsampling, PixelShuffle simultaneously compresses the channel dimension of deep features, which significantly reduces the total number of channels involved in the subsequent fusion stage.A subsequent 1×1 convolution is applied to adjust the number of channels, ensuring that the high-level features are fully aligned with the low-level ones in both resolution and dimensionality. The formula is as follows:


$${F_{h}}^{\prime }=\delta \left({\text{Conv}}_{3\times 3}\left(Up\left(F_{h}\right)\right)\right),$$ (2)

where $F_{h}$ denotes the concatenated high-level features, Up(·) represents the pixelshuffle upsampling operation, and ${F_{h}}^{\prime }$ refers to the upsampled and channel-aligned high-level feature map, which serves as the input for the subsequent attention-based fusion.

Subsequently, CLSCM applies attention-driven weighted enhancement to both the original low-level features and the aligned high-level features, where the attention responses are interpreted as compensation strength rather than mere feature-importance scores. For the high-level feature branch, the module integrates a spatial attention gating unit (SAGU) and a channel squeeze-and-excitation unit (CSEU) to dynamically modulate semantic responses via dual attention mechanisms in spatial and channel dimensions. Specifically, the input feature is first projected through a 1×1 convolution to produce three feature groups: Query (Q), Key (K), and Value (V). Among them, the Q and K branches encode semantic relevance from spatial and channel perspectives, respectively, while the V branch preserves the original high-level semantic content. To enhance discriminative capability, Q and K are further refined by the SAGU and CSEU to enhance spatial saliency and channel-wise selectivity. The enhanced Q and K are then combined to form a semantic similarity function Sim(Q,K),which explicitly models the necessity and strength of semantic compensation at each spatial location and channel. When element-wise multiplied with V,the resulting response adaptively enhances or suppresses semantic injection according to scene complexity, object scale, and boundary ambiguity. Finally, aa convolution and dropout operation are applied to further improve robustness and mitigate overfitting, producing discriminative high-level semantic feature weights. The relevant formulas are as follows:


$$Q,K,V=Split(Conv_{1\times 1}({F_{h}}^{\prime }),dim=1),$$ (3)

$$E_{s}=\sigma \left(Conv_{1\times 1}\left(\delta \left(ℬ\left(Conv_{3\times 3}\left(x\right)\right)\right)\right)\right)⊙x,$$ (4)

$$E_{c}=\sigma \left(Conv_{1\times 1}\left(Conv_{1\times 1}\left(x\right)\right)\right)⊙x,$$ (5)

Sim(Q,K)=Φ(Q)+Φ(K), (6)

$${F_{h}}^{\prime \;\prime }=Dropout\left(Conv_{3\times 3}\left(Conv_{3\times 3}\left(Sim\left(Q,K\right)⊙V\right)\right)\right),$$ (7)

where ⊙ denotes the Hadamard product, σ represents the sigmoid activation function, denotes the ReLU activation function, δ denotes the ReLU activation function, and ℬ refers to batch normalization, $E_{s}$, $E_{c}$ corresponds to the outputs of the SAGU and the CSEU, respectively. Stacking the two enables joint spatial and channel attention modeling, yielding a fused, attention-enhanced feature representation, denoted as Φ(·). ${F_{h}}^{\prime \;\prime }$ represents the refined high-level semantic weights.

In the low-level branch, the original low-level featurefirst normalized using layer normalization and passed through a 3×3 depthwise separable convolution to generate a spatial attention map (Att).This map explicitly controls where semantic information should be absorbed. Simultaneously, a 1×1 convolution is applied to produce the value tensor (V). Then, Hadamard product (Att⊙V) is used to explicitly weight the key regions. Subsequently, a 1×1 convolution is applied to align the number of channels, resulting in the spatially optimized low-level feature ${F_{l}}^{\prime \;\prime }$. This bidirectional design ensures that low-level features do not passively receive semantic compensation but selectively accept it only when necessary, thereby preventing over-smoothing and preserving fine-grained structural details.The relevant formulas are as follows:


$${F^{\prime }}_{l}=LayerNorm\left(F_{l}\right),$$ (8)

$$Att=Conv_{3\times 3}\left(GELU\left(Conv_{1\times 1}\left({F^{\prime }}_{l}\right)\right)\right),\text{\textbackslash{}hspace\{0.17em}\}V=Conv_{1\times 1}\left({F^{\prime }}_{l}\right),$$ (9)

$${F_{l}}^{\prime \;\prime }=Conv_{1\times 1}\left(\left(Att⊙V\right)\right),$$ (10)

where ⊙ denotes the Hadamard product, GELU denotes the GELU activation function, and layerNorm refers to layer normalization.

Finally, the weighted high-level semantic features are element-wise multiplied with the spatially optimized low-level features. The result is then passed through a convolutional layer to obtain the fused feature $F_{fused}$, enabling dynamic and hierarchical integration of fine-grained spatial details and semantic context. While the above compensation process effectively aligns semantics across different feature levels, its receptive field remains locally constrained, which limits the modeling of global context and long-range dependencies. To address this limitation, CLSCM further integrates a Large Selective Kernel (LSK) module, which expands the perceptual range of the compensated features by combining multi-scale depthwise separable convolutions with an attention-based selection mechanism [46]. This design enhances the representation of small objects, blurred boundaries, and fine structures under complex backgrounds, thereby improving robustness and semantic consistency in challenging scenes.. Specifically, the input $F_{fused}\in {\mathbb{R}}^{C\times H\times W}$ is first processed by two depthwise separable convolutions with different kernel sizes to extract diverse features $F_{\text{1}}$ and $F_{\text{2}}$, which are then dimensionally reduced via 1×1 convolutions and concatenated into $F_{c}$. After applying average and max pooling to $F_{c}$, the pooled features are concatenated and passed through a convolution to generate attention weights $A=\left[A_{\text{1}},A_{\text{2}}\right]$. These weights guide the fusion of $F_{\text{1}}$ and $F_{\text{2}}$, producing $F_{\text{att}}$, which is finally projected back to the original channel size and added to $F_{fused}$, yielding the residual-enhanced output $F_{out}$. This module avoids the sampling flaws of dilated convolutions, expands the receptive field, and improves robustness to blurred boundaries, small objects, and background noise. It complements the spatial modeling limitations of CLSCM, enhancing segmentation accuracy and generalization in complex urban–rural scenes.The corresponding formulas are defined as follows:


$$F_{\text{1}}=DSConv_{5\times 5}\left(F_{fused}\right),\text{\textbackslash{}hspace\{0.17em}\}F_{\text{2}}=DSConv_{7\times 7}\left(F_{\text{1}}\right)$$ (11)

$$F_{c}=Concat\left(Conv_{1\times 1}\left(F_{\text{1}}\right),Conv_{1\times 1}\left(F_{\text{2}}\right)\right),$$ (12)

$$A=\sigma \left(Conv_{7\times 7}\left(Concat\left(AvgPool\left(F_{c}\right),MaxPool\left(F_{c}\right)\right)\right)\right),$$ (13)

$$F_{att}=A_{\text{1}}\cdot Conv_{1\times 1}\left(F_{\text{1}}\right)+A_{\text{2}}\cdot Conv_{1\times 1}\left(F_{\text{2}}\right),$$ (14)

$$F_{out}=F_{fused}\cdot Conv_{1\times 1}\left(F_{att}\right),$$ (15)

where σ represents the sigmoid activation function, DSConv(·) denotes the depthwise separable convolutions.

### 3.5. Auxiliary boundary supervision module (ABSM)

**Fig 5:**
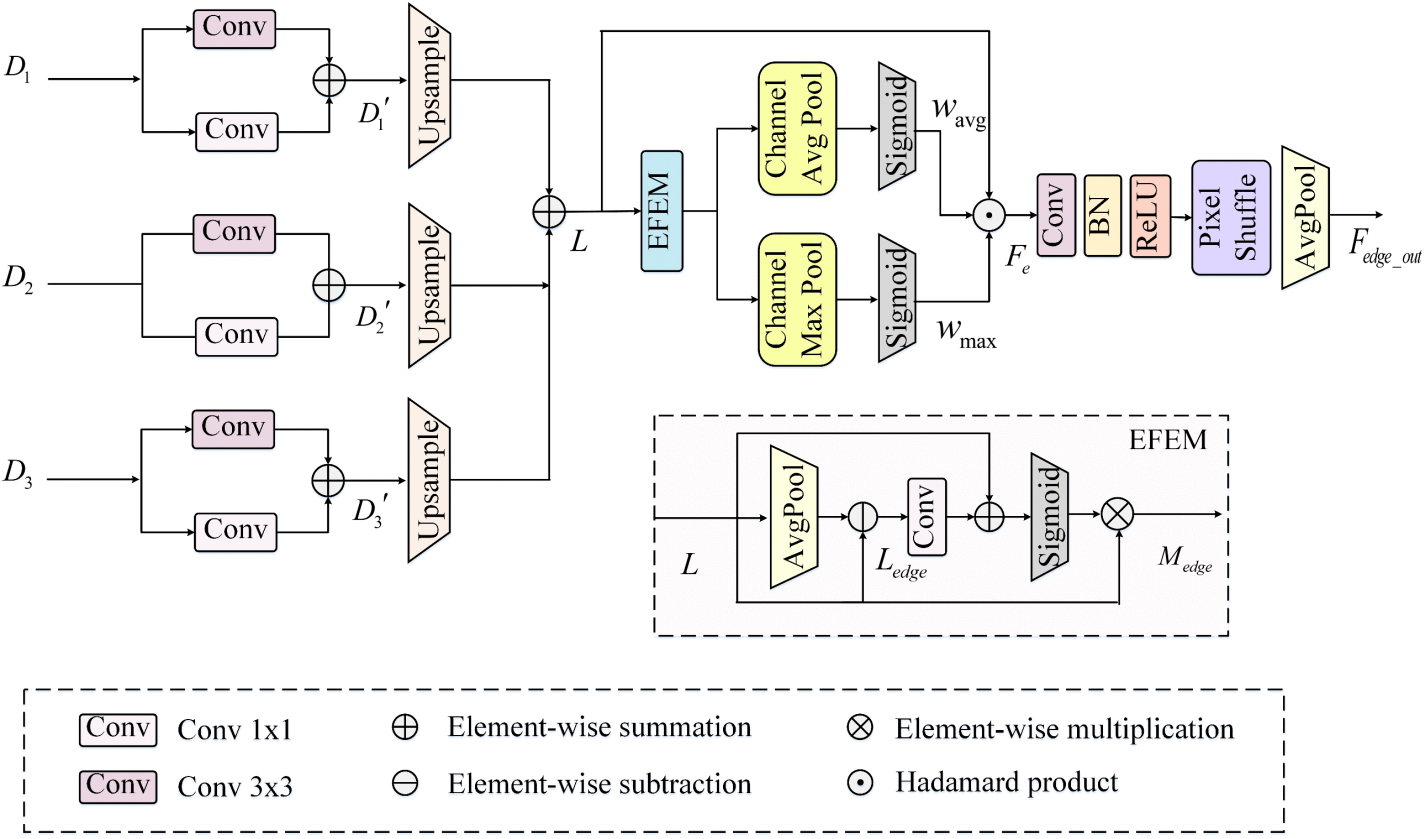
The detailed architecture of the proposed ABSM.

In urban–rural remote sensing image semantic segmentation, accurate identification of object boundaries is crucial for achieving high segmentation performance. To address this, in the proposed BGSC-Net, we propose a decoder-driven auxiliary boundary supervision module (ABSM), which integrates multi-level decoder features, local detail enhancement, and explicit boundary modeling within an auxiliary branch, the detailed structure is illustrated in Fig 5. Moreover, a boundary supervision strategy based on a dedicated loss function is introduced to effectively strengthen boundary representation and improve segmentation accuracy in complex scenes.

As shown in the Fig 5, the ABSM processes multi-level decoder features ($D_{\text{1}}$, $D_{\text{2}}$, $D_{\text{3}}$) through two parallel branches: a 3×3 convolution for contextual cues and a 1×1 convolution for fine details. The outputs are normalized (batch normalization) and activated (ReLU6 activation function), then fused and upsample via bilinear interpolation. These are added to higher-resolution features from earlier stages, constructing a hierarchical detail enhancement path that effectively integrates multi-scale information, strengthens boundary representation, and improves both segmentation accuracy and robustness. The relevant formulas are as follows:


$${D^{\prime }}_{i}=\delta \left(ℬ\left(Conv_{3\times 3}\left(D_{i}\right)\right)\right)+\delta \left(ℬ\left(Conv_{1\times 1}\left(D_{i}\right)\right)\right),$$ (16)

$$L=\sum_{i=1}^{N}Upsample({D_{i}}^{\prime }),$$ (17)

where δ denotes the ReLU activation function, ℬ represents the batch normalization, idenotes the index of the decoder stage, N indicates the number of decoder layers involved in the multi-scale fusion, and L represents the fused feature after local detail enhancement.

The core objective of the edge-aware feature enhancement module (EFEM) is to explicitly extract edge information and fuse it with the original features, thereby enhancing the model’s perception of boundary regions and structural details. This facilitates improved accuracy and continuity in boundary segmentation within remote sensing images. As illustrated in the Fig 5, the processing flow of EFEM is as follows: first, a pooling operation is applied to the input features to extract low-frequency background information. The pooled result is subtracted element-wise from the original features to produce an edge-enhanced feature map $L_{\text{edge}}$, which highlights abrupt boundary changes. This map is then processed by a 1×1 convolution, batch normalization, and sigmoid activation to generate an edge attention map, which is used to perform weighted fusion with the original features. An additional attention mechanism is then applied to further enhance the responses of salient regions while suppressing redundant information, resulting in the preliminary enhanced feature map $M_{\text{edge}}$. The relevant formulas are as follows:


$$L_{edge}=L-AvgPool\left(L\right),$$ (18)

$$M_{edge}=\sigma \left(L+ℬ\left(Conv_{1\times 1}\left(L_{edge}\right)\right)\right)\cdot L,$$ (19)

where δ represents the sigmoid activation function, ℬ represents the batch normalization.To further strengthen the model’s focus on key regions, the ABSM computes both average and maximum responses across the channel dimension, producing two complementary spatial attention maps $w_{\text{avg}}$ and $w_{\text{max}}$. These attention maps enable a more comprehensive exploration of salient regions. Finally, the two attention maps are element-wise multiplied with the previously obtained feature L to perform spatial enhancement, resulting in the edge-enhanced feature map $F_{e}$. The formulas are as follows:


$$w_{avg}=\sigma \left(\frac{\text{1}}{C}\sum_{c=1}^{C}\overset{\left(c\right)}{\underset{edge}{M}}\right),\text{\textbackslash{}hspace\{0.17em}\}w_{max}=\sigma \left({max}_{c=1,\ldots ,C}\overset{\left(c\right)}{\underset{edge}{M}}\right),$$ (20)

$$F_{e}=L\cdot w_{avg}\cdot w_{max}\text{,}$$ (21)

where σ represents the sigmoid activation function, C represents the number of channels. After the above enhancement, the edge feature map is further processed to generate a class-wise intermediate prediction suitable for auxiliary supervision. A 1×1 convolution is first applied to adjust the feature channels. This is followed by a pixelshuffle upsampling operation, which spatially redistributes semantic cues originally encoded in the channel dimension to the spatial domain. Unlike traditional point-wise predictions, this enables each pixel to integrate semantic information from its surrounding region, facilitating a shift from isolated prediction to local-area fusion.

To further enhance robustness, average pooling is applied to smooth the locally fused features, effectively suppressing noise and reducing redundancy. This combination of pixelshuffle upsampling and average pooling not only strengthens semantic continuity across boundaries but also significantly improves the stability and clarity of edge prediction, especially in complex scenarios with noisy backgrounds or adjacent categories. Finally, bilinear interpolation is used to upsample the edge map to match the spatial resolution of the main segmentation output. The corresponding computation is formulated as follows:


$$F_{edge\_out}=AvgPool\left(PixelShuffle\left(\delta \left(ℬ\left(Conv_{1\times 1}\left(F_{e}\right)\right)\right)\right)\right),$$ (22)

$$F_{resized}=Interp\left(F_{edge\_out},size=(H,W)\right),$$ (23)

where δ represents the sigmoid activation function, ℬ represents the batch normalization. Interp(·) denotes bilinear interpolation upsampling.

The resulting prediction forms an auxiliary supervision branch, whose loss is combined with the main decoding loss using a predefined weight during training (as detailed in Section 3.6). This auxiliary branch provides complementary gradients that guide the backbone network in focusing on boundary regions and capturing fine structures. By explicitly modeling edge semantics, it mitigates boundary detail loss and enhances segmentation accuracy and robustness, particularly in challenging urban–rural scenes with blurred edges, dense targets, or adjacent classes. It also improves the structural consistency of predicted masks, boosting real-world applicability.

### 3.6. Loss Function

To fully leverage the critical role of the decoder-driven auxiliary boundary supervision module (ABSM) in edge enhancement, and to guide the model toward precise boundary detection in complex urban–rural remote sensing scenes, we adopt a multi-task joint loss framework. The total loss combines the main segmentation loss and an auxiliary edge loss derived from the ABSM’s output, providing explicit supervision for boundary regions. This effectively addresses common challenges such as blurred edges, dense objects, and adjacent classes. To support this framework, we employ two representative loss functions-Cross-Entropy Loss and Dice Loss-which offer complementary strengths in pixel-level semantic segmentation. Cross-Entropy Loss quantifies the difference between predicted probabilities and one-hot encoded labels, ensuring accurate pixel-wise classification. Dice Loss, on the other hand, highlights the spatial overlap between predictions and ground truth, improving segmentation performance, especially in imbalanced scenarios. The formulas are described as follows:


$${ℒ}_{ce}=-\frac{\text{1}}{N}\sum_{n=1}^{N}\sum_{k=1}^{K}\overset{\left(n\right)}{\underset{k}{y}}log\left(\overset{\left(n\right)}{\underset{k}{\hat{y}}}\right),$$ (24)

$${ℒ}_{dice\;}=1-\frac{\text{2}}{N}\sum_{n=1}^{N}\sum_{k=1}^{K}\frac{\overset{\left(n\right)}{\underset{k}{y}}\overset{\left(n\right)}{\underset{k}{\hat{y}}}}{\overset{\left(n\right)}{\underset{k}{y}}+\overset{\left(n\right)}{\underset{k}{\hat{y}}}},$$ (25)

where N is the number of samples, K is the number of classes. The variables $\overset{\left(n\right)}{\underset{k}{y}}$ and $\overset{\left(n\right)}{\underset{k}{y}}$ represent the confidence of the k -th label and the model’s prediction for the n -th data sample, respectively.

In the proposed BGSC-Net, we design an auxiliary boundary loss based on Cross-Entropy Loss, which is computed from the edge feature maps produced by the ABSM. The main segmentation loss is formulated as a combination of Dice Loss and Cross-Entropy Loss, aiming to balance pixel-level classification accuracy and shape consistency. The total loss is defined as a weighted sum of the main segmentation loss and the auxiliary boundary loss. The formulas are described as follows:


$${ℒ}_{aux}=-\frac{\text{1}}{N}\sum_{n=1}^{N}\sum_{k=1}^{K}\overset{\left(n\right)}{\underset{k}{y}}log\left(\overset{\left(n\right)}{\underset{k}{d}}\right),$$ (26)

$${ℒ}_{main\;}={ℒ}_{ce}+{ℒ}_{dice\;},$$ (27)

$${ℒ}_{total\;}={ℒ}_{main\;}+\lambda \cdot {ℒ}_{aux\;},$$ (28)

where $\overset{\left(n\right)}{\underset{k}{d}}$ denotes the integrated features from intermediate decoder blocks, and λ represents the weight of the auxiliary loss, which is set to 0.4 in our implementation.

This design provides additional boundary guidance to the main segmentation branch while enhancing the overall learning capacity and segmentation performance of the model through the complementary nature of multi-task optimization. The benefits are particularly evident in complex urban–rural remote sensing scenarios, where the model demonstrates superior performance in segmenting small objects and fine-grained boundaries.

## 4. Experimental results and analyses

### 4.1. Datasets

To evaluate the effectiveness and generalization capability of the proposed BGSC-Net, we conducted extensive comparative and ablation experiments on four public datasets as well as the self-constructed MSFSD. This section begins by introducing the characteristics of the datasets, followed by a description of the experimental settings and evaluation metrics. We then present comparative results against state-of-the-art models to assess overall segmentation performance, and ablation studies to quantify the contribution of each network component. Finally, we analyze the model’s computational complexity to evaluate its practical efficiency.

Potsdam [47]: The dataset consists of remote sensing images (5 cm GSD) tiles, each sized 6000 × 6000 pixels, and shares the same class structure as Vaihingen. Besides RGB bands, DSM and NDSM are also provided, but only the RGB channels are used in our experiments. According to the official split, 14 tiles (IDs:2_13, 2_14, 3_13, 3_14, 4_13, 4_14, 4_15, 5_13, 5_14, 5_15, 6_13, 6_14, 6_15, 7_13) are used for testing, 2_10 for validation, and the remaining 22

tiles for training (excluding 7_10 due to annotation errors). All original images are cropped into 1024 × 1024 pixel patches.

Vaihingen [47]: The dataset includes 33 remotcankaoe sensing image (9 cm GSD) tiles, with an average size of 2494 × 2064 pixels, and provides near-infrared, red, green spectral bands along with DSM/NDSM data. The ground truth includes five foreground classes (impervious surface, building, low vegetation, tree, car) and one background class. In our experiments, only spectral (RGB) images are used, discarding the DSM/NDSM information. We follow a custom split: 17 images (IDs: 2, 4, 6, 8, 10, 12, 14, 16, 20, 22, 24, 27, 29, 31, 33, 35, 38) are used for testing, ID 30 for validation, and the remaining 15 images for training. All images are cropped into 1024 × 1024 patches for network training.

LoveDA [48]: The dataset comprises 5987 high-resolution optical remote sensing images (1024 × 1024 pixels at 0.3 m GSD), covering both urban and rural scenes with seven land cover categories (building, road, water, barren, forest, farmland, background) (Wang et al., 2021a). The dataset includes 2522 images for training, 1669 for validation, and 1796 for testing. Due to the presence of multi-scale objects, complex backgrounds, and class imbalance, LoveDA poses a significant challenge for models in terms of generalization and fine-grained segmentation capabilities.

UAVid [49]: The dataset is a high-resolution UAV image dataset for semantic segmentation, featuring two ultra-high resolutions (3840 × 2160 and 4096 × 2160) and covering eight land cover categories, including building, road, tree, low vegetation, moving car, static car, human, and clutter. The dataset presents considerable challenges due to its fine-grained spatial textures, diverse object scales, and complex scene variations. It consists of 420 images captured from 42 flight sequences, with 200 images for training, 70 for validation, and 150 for testing. In this study, each image is padded and split into eight patches of size 1024 × 1024 pixels to meet the input requirements of the network.

MSFSD: This dataset is a high-resolution UAV image dataset for fine-grained mangrove species segmentation, featuring a ground resolution of 2.5 cm/pixel and covering five representative mangrove species: Aegiceras corniculatum, Avicennia marina, Bruguiera gymnorrhiza, Rhizophora stylosa, and Kandelia obovata. The dataset presents considerable challenges due to the high visual similarity between species, complex canopy structures, and substantial seasonal variations. It consists of 2,640 annotated image patches of size 512 × 512

pixels extracted from 12,560 original UAV images, with 1,584 patches for training, 528 for validation, and 528 for testing. All images were acquired at a low altitude of 10 meters above ground level with a flight speed of 3 m/s using a DJI Mavic 3E UAV equipped with a high-resolution RGB camera over the Guangxi Beihai Shakou Mangrove National Nature Reserve (21°28’N, 109°37’E) from September 2024 to June 2025, ensuring comprehensive coverage of seasonal ecological changes.

### 4.2. Experimental setting

The experiments were conducted on a server running Ubuntu 18.04, equipped with an NVIDIA GeForce RTX 4090 GPU (24 GB VRAM). The development environment was based on Python 3.8, using PyTorch 2.0.0 + cu118 as the deep learning framework. The AdamW optimizer was employed, along with a CosineAnnealingLR scheduler for smooth learning rate annealing. The initial learning rate was set to 1 × 10 ⁻ ³ for the back-bone network and 9 × 10 ⁻ ³ for the remaining parts, with a weight decay of 0.01. All exper-iments were trained with a batch size of 8. For the UAVid dataset, input images were resized to 1024 × 1024, and the model was trained for 40 epochs. For the ISPRS Potsdam and Vaihingen datasets, the images were randomly cropped to 512 × 512 and trained for 45 and 105 epochs, respectively. The LoveDA dataset also used 512 × 512 patches and was trained for 45 epochs. For the MSFSD, the model was trained for 105 epochs using the original 512 × 512 patches to ensure adequate convergence on this fine-grained species segmentation task. During training, various data augmentation techniques-such as ran-dom rotation, flipping, brightness/contrast adjustment, cropping, scaling, and sharpen-ing were employed to enhance robustness to complex boundaries and diverse scenes. This setup enables the model to effectively learn fine-grained segmentation features while ensuring training efficiency and optimal resource utilization.

### 4.3. Evaluation measure

To comprehensively evaluate the segmentation performance of the model, we adopt overall accuracy (OA), mean intersection over union (mIoU), and mean F1-score (mF1) as the primary evaluation metrics. The specific calculation formulas for these metrics are presented as follows:


$$OA=\frac{\sum_{k=1}^{K}TP_{k}}{\sum_{k=1}^{K}\left(TP_{k}+FP_{k}+TN_{k}+FN_{k}\right)}\text{,}$$ (29)

$$mIoU=\frac{\text{1}}{K}\sum_{k=1}^{K}\frac{TP_{k}}{TP_{k}+FP_{k}+FN_{k}},$$ (30)

$$precision\;k=\frac{TP_{k}}{TP_{k}+FP_{k}},\text{\textbackslash{}hspace\{0.17em}\}recall\;k=\frac{TP_{k}}{TP_{k}+FN_{k}},$$ (31)

$$mF1=\frac{\text{1}}{K}\sum_{k=1}^{K}F{\text{1}}_{k}=\frac{\text{1}}{K}\sum_{k=1}^{K}\frac{2\times \;precision\;k\times \;recall\;k}{\;precision\;k+\;recall\;k},$$ (32)

where $TP_{k}$, $FP_{k}$, $TN_{k}$, and $FN_{k}$ denote the number of true positives, false positives, true negatives, and false negatives for class k, respectively. These metrics provide a comprehensive assessment of segmentation performance in terms of both pixel-wise accuracy and class-wise discrimination.

To further quantitatively evaluate the boundary preservation ability of different models, we additionally adopt three boundary-aware metrics, including Boundary IoU (B-IoU), Boundary F-score (B-F1), and trimap F-score (T-F1). The formulas are described as follows:


B(P)=Boundary(P),\hspace{0.17em}B(G)=Boundary(G) (33)

$$\hat{B}\left(P\right)=Dilate\left(B\left(P\right),r\right),\text{\textbackslash{}hspace\{0.17em}\}\hat{B}\left(G\right)=Dilate\left(B\left(G\right),r\right)$$ (34)

$$BIoU=\frac{\left|\hat{B}\left(P\right)\cap \hat{B}\left(G\right)\right|}{\left|\hat{B}\left(P\right)\cup \hat{B}\left(G\right)\right|},\text{\textbackslash{}hspace\{0.17em}\}mBIoU=\frac{\text{1}}{K}\sum_{k=1}^{K}BIoU,$$ (35)

where B(P) and B(G) denote the boundary maps extracted from the prediction P and ground truth G, respectively. $\hat{B}\left(P\right)$ and $\hat{B}\left(G\right)$ represent the dilated boundary regions with a radius r. BIoU measures the intersection-over-union between the dilated predicted and ground-truth boundaries, and mBIoU is obtained by averaging over all K classes.This metric evaluates the boundary overlap between prediction and ground truth with tolerance to small localization errors.


$$P_{bf}=\frac{\left|B\left(P)\cap \hat{B}(G\right)\right|}{\left|B\left(P\right)\right|},R_{bf}=\frac{\left|B\left(G)\cap \hat{B}(P\right)\right|}{\left|B\left(G\right)\right|}$$ (36)

$$BF1=\frac{2P_{bf}R_{bf}}{P_{bf}+R_{bf}}=\frac{2\left|B\left(P)\cap \hat{B}(G\right)\right|}{\left|B\left(P\right)\right|+\left|B\left(G\right)\right|},mBF1=\frac{\text{1}}{K}\sum_{k=1}^{K}BF1$$ (37)

where $P_{bf}$ and $R_{bf}$ denote the boundary precision and recall. $P_{bf}$ measures the proportion of predicted boundary pixels that fall within the dilated ground-truth boundary region, while $R_{bf}$ indicates the proportion of ground-truth boundary pixels that are correctly matched by the predicted boundaries. the intersection-over-union between the dilated predicted and ground-truth boundaries, BF1 is the harmonic mean of $P_{bf}$ and $R_{bf}$, and mBF1 is computed by averaging over all classes.This metric reflects the accuracy and completeness of boundary localization.


T=Dilate(G,r)−Erode(G,r), (38)

$$P_{tri}=\frac{\left|B(P)\cap \hat{B}(G)\cap T\right|}{\left|B(P)\cap T\right|},R_{tri}=\frac{\left|B(G)\cap \hat{B}(P)\cap T\right|}{\left|B(G)\cap T\right|}$$ (39)

$$TF1=\frac{2P_{tri}R_{tri}}{P_{tri}+R_{tri}}=\frac{2|B(P)\cap \hat{B}(G)\cap T|}{\left|B(P)\cap T|+|B(G)\cap T\right|},mTF1=\frac{\text{1}}{K}\sum_{k=1}^{K}TF1,$$ (40)

where T denotes the trimap region constructed by dilating and eroding the ground-truth mask with radius r. $P_{tri}$ and $R_{tri}$ represent the precision and recall computed within the trimap region, respectively. TF1 is the harmonic mean of $P_{tri}$ and $R_{tri}$, and mTF1 is com-puted by averaging over all classes.This metric evaluates the segmentation accuracy spe-cifically in boundary-adjacent regions.

### 4.4. Comparative Experiments

To validate the effectiveness and generalization capability of the proposed method, extensive experiments and comparative evaluations were conducted on four established remote sensing semantic segmentation datasets and our self-constructed MSFSD. The experiments included classic models such as ABCNet [50], MANet [51], and A2-FPN [29]; advanced CNN-based encoder methods including MAResU-Net [30], SFFNet [52], CMTFNet [25], and DecoupleNet [53]; representative large-scale Transformer-based encoder models such as DC-Swin [54]; as well as networks employing hybrid encoder-decoder architectures like UNetFormer [10], SFANet [11]and BAFormer [24]. For a more comprehensive comparison, we additionally evaluate a recently proposed state-space model based method, UrbanSSF [28], which adopts a Mamba-style selective state-space modeling mechanism to model long-range dependencies. Moreover, two recently proposed approaches, AFENet [55] and BiCoR-Seg [56], are also included as comparison baselines. All models were trained and tested under the same hardware and software conditions to ensure fairness, data consistency, and reliable comparison. Detailed experimental results are presented in the tables and visual comparison figures. All models were trained and tested under the same hardware and software conditions to ensure fairness, data consistency, and reliable comparison. Detailed experimental results are presented in the tables and visual comparison figures.

#### 4.4.1. Experimental results on the Potsdam dataset.

Table 1 summarizes the segmentation performance of different models on the Potsdam dataset. Overall, BGSC-Net achieves the best performance across key metrics, with a MeanF1 of 93.24%, OA of 91.95%, and mIoU of 87.57%, significantly outperforming other competing models. This clearly demonstrates its advantages in segmenting complex remote sensing scenes. In particular, it achieves IoU scores of 94.24% for buildings, 89.50% for impervious

**Table 1:** Segmentation results of different models on the Potsdam dataset. The values in bold represent the top-performing metrics in the table.

Methods	Per-Class IoU(%)					MeanF1(%)	OA(%)	mIoU(%)
	Imp.surf	Building	Low.veg	Tree	Car			
MANet [51]	85.39	88.66	74.58	77.50	91.59	90.89	89.31	83.54
ABCNet [50]	88.01	92.54	76.56	78.35	91.62	91.99	90.74	85.41
MAResU-Net [30]	87.00	91.66	75.94	77.83	90.98	91.56	90.35	84.68
A2-FPN [29]	87.98	92.18	76.69	79.85	91.31	92.12	90.97	85.60
DC-Swin [54]	89.10	93.95	78.38	80.19	91.64	92.73	91.59	86.65
UNetFormer [10]	88.48	92.44	77.86	79.81	92.81	92.51	91.25	86.28
SFFNet [52]	87.99	92.83	76.99	79.52	91.71	92.23	90.99	85.81
SFANet [11]	88.98	94.04	78.39	80.81	92.33	92.87	91.75	86.91
AFENet [55]	89.15	93.65	78.50	80.35	92.78	92.93	91.79	86.95
BiCoR-Seg [56]	89.29	93.79	78.65	80.47	92.92	92.94	91.76	87.02
UrbanSSF [28]	89.57	**94.57**	78.36	80.57	92.86	93.02	91.86	87.19
BGSC-Net	**89.50**	94.24	**78.96**	**81.01**	**94.11**	**93.24**	**91.95**	**87.57**

surfaces, and 94.11% for cars, markedly surpassing mainstream methods such as MANet [51], MAResU-Net [30], and UNetFormer [10]. In addition, compared to representative Transformer-based and hybrid models such as DC-Swin [54] and SFANet [11],BGSC-Net also exhibits superior segmentation accuracy in particularly challenging categories, including small objects (e.g., cars) and semantically similar background classes (e.g., low vegetation and trees), which are often difficult to distinguish due to their spectral and structural similarities. This reflects the model’s strong capacity for fine-grained feature discrimination and robust generalization in complex, multi-scale remote sensing environments. Furthermore, BGSC-Net consistently outperforms recently proposed methods, including the state-space model based UrbanSSF [28] and the newly proposed AFENet [55] and BiCoR-Seg [56]. Specifically, compared with UrbanSSF [28], BGSC-Net achieves improvements of +0.22% in mF1 and +0.38% in mIoU, indicating that although state-space modeling effectively captures long-range dependencies, our method further benefits from explicit multi-scale feature fusion and spatial attention. For AFENet [55] and BiCoR-Seg [56], BGSC-Net also demonstrates clear advantages on challenging categories such as cars and low vegetation. For example, BGSC-Net achieves 94.11% IoU on cars, surpassing AFENet [55] (92.78%) and BiCoR-Seg [55] (92.92%). These results further verify the strong capability of our model in distinguishing small objects and semantically similar classes.

**Fig 6:**
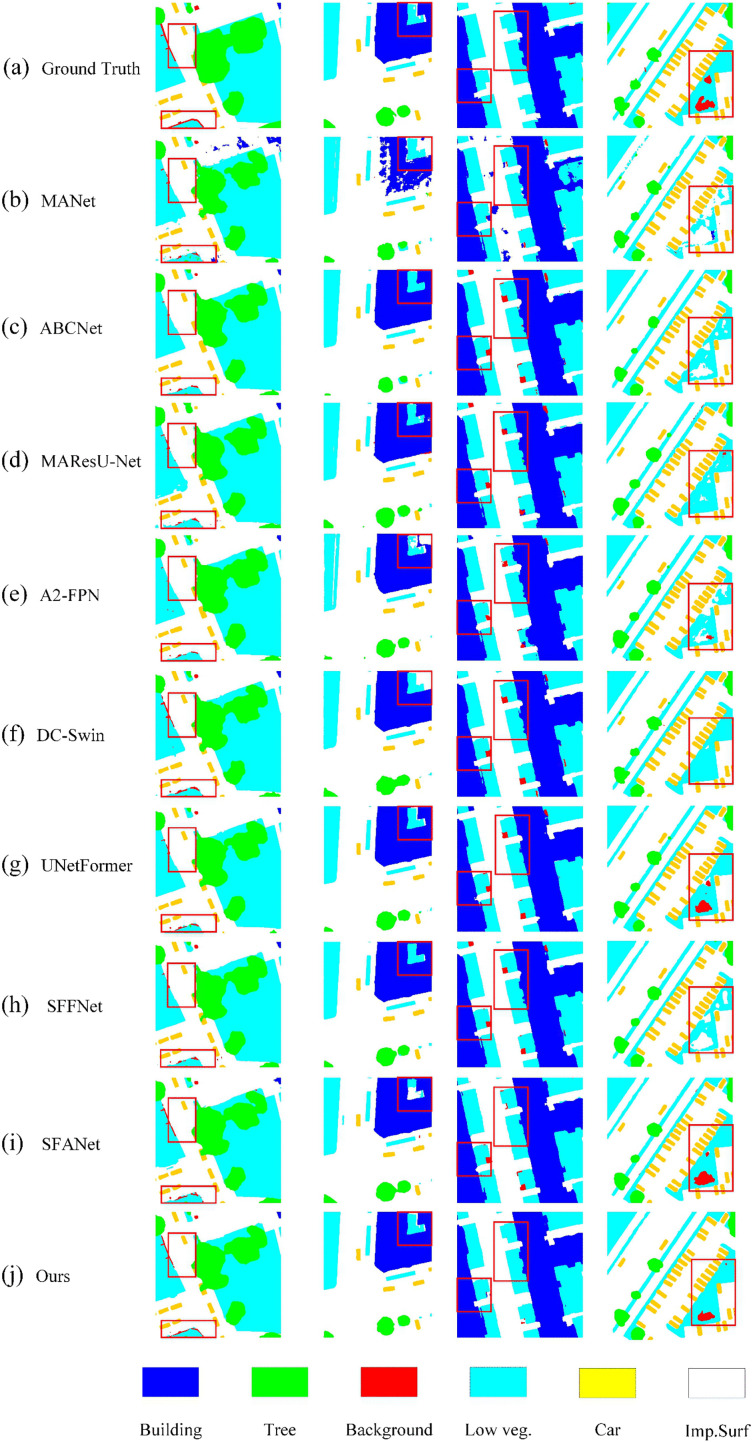
Visualization results of comparative experiments on the Potsdam dataset.

Fig 6 illustrates the visual segmentation results of BGSC-Net on the Potsdam dataset, highlighting its superiority in complex urban scenes. In the first and fourth columns, BGSC-Net accurately outlines building boundaries despite dense surrounding vegetation, outperforming other models that exhibit discontinuities, class confusion, and artifacts. Moreover, BGSC-Net effectively distinguishes between visually similar categories such as low vegetation and impervious surface, maintaining clear and consistent class boundaries regardless of shape complexity. The second and third examples highlight its fine-grained segmentation capability in multi-class interwoven regions, where it preserves both structural continuity of impervious surface and edge integrity of buildings. In small-object segmentation, BGSC-Net excels in delineating vehicles with smooth and coherent contours, effectively mitigating typical issues like fragmented edges and jagged outlines, thus demonstrating its strong capacity for detail preservation and boundary refinement.

To quantitatively support the visual observations in Fig 6, we further report boundary-related evaluation results on the Potsdam dataset in Table 2, including Boundary-F1, trimap F-score, and Boundary-IoU. BGSC-Net achieves the best performance on all three metrics, with scores of 62.37%, 73.22%, and 37.94%, respectively, clearly surpassing all competing methods.These results are highly consistent with the qualitative analysis. In particular, the superior Boundary-F1 and Boundary-IoU indicate that BGSC-Net produces more accurate and complete object contours, especially in complex urban scenes where buildings are tightly surrounded by vegetation. Compared with CNN-based models (e.g., MANet [51] and MAResU-Net [30]) and Transformer-based architectures (e.g., DC-Swin [54] and UNetFormer [10]), BGSC-Net demonstrates significantly improved boundary localization abil-ity, effectively reducing discontinuities and boundary artifacts. Moreover, BGSC-Net also outperforms recently proposed models such as UrbanSSF [28], AFENet [55], and BiCoR-Seg [56] on all boundary metrics, showing that explicit boundary supervision is more effective than solely relying on long-range dependency modeling. Notably, the improvement is particularly evident for small objects such as cars. As observed in Fig. 6, vehicles often suffer from fragmented edges in existing methods, while BGSC-Net generates smoother and more coherent contours. This is quantitatively confirmed by the higher Boundary-F1 and Boundary-IoU scores, demonstrating the model’s strong capability in preserving fine details and accurately delineating small-scale targets.

**Table 2:** Boundary accuracy comparison of different models on the Potsdam dataset. The values in bold represent the top-performing metrics in the table.

Methods	Per-Class B-IoU/T-F1(%)					mB-F1(%)	mT-F1(%)	mB-IoU(%)
	Imp.surf	Building	Low.veg	Tree	Car			
MANet [51]	31.36/72.79	27.85/72.42	28.47/67.90	21.58/60.93	52.07/79.39	54.30	70.71	32.26
ABCNet [50]	34.45/72.75	32.30/73.75	29.39/66.74	21.21/59.93	53.88/79.65	55.13	70.56	34.24
MAResU-Net [30]	36.72/73.88	35.25/74.63	29.59/68.47	21.92/62.44	54.11/79.96	57.71	71.87	35.51
A2-FPN [29]	36.63/7361	30.24/73.92	28.93/67.53	21.07/63.26	50.62/78.56	55.16	71.37	33.49
DC-Swin [54]	38.08/74.72	37.63/76.29	30.81/69.15	23.28/63.84	54.46/79.96	59.38	72.79	36.85
UNetFormer [10]	36.67/74.12	35.19/75.32	29.84/68.52	22.37/62.57	54.80/80.40	58.49	72.18	35.77
SFFNet [52]	36.60/73.13	32.67/73.98	28.36/69.32	21.04/61.85	42.51/78.45	56.00	71.34	34.23
SFANet [11]	38.84/74.41	**37.99**/75.62	31.29/68.90	22.45/**65.47**	56.09/80.34	60.41	72.94	37.33
AFENet [55]	38.53/74.25	36.63/75.08	30.35/68.34	23.51/62.82	55.11/79.68	60.11	72.03	36.78
BiCoR-Seg [56]	38.98/74.84	37.08/75.67	30.80/68.93	23.96/63.41	55.56/80.12	93.02	72.59	37.25
UrbanSSF [28]	**39.16/75.02**	37.26/75.85	30.98/69.11	24.14/63.59	55.74/80.30	61.06	72.77	37.45
BGSC-Net	38.89/74.29	37.76**/76.27**	**31.64/69.95**	**24.28/**64.37	**57.04/80.62**	**62.37**	**73.22**	**37.94**

#### 4.4.2. Experimental results on the Vaihingen dataset.

Table 3 summarizes the segmentation performance comparison of various models on the Vaihingen dataset. The experimental results demonstrate that the proposed model achieves outstanding performance, with a Mean F1 score of 92.08%, Overall Accuracy (OA) of 93.80%, and mean Intersection over Union (mIoU) of 85.61%, all surpassing existing methods. BGSC-Net exhibits strong fine-grained segmentation capabilities across all classes, notably achieving an IoU of 83.25% for the car category, significantly outperforming other compared models. For the impervious surface and building classes, the model attains IoUs of 94.25% and 92.83%, respectively, substantially exceeding most competitors. Although some models (e.g., BiCoR-Seg [56], UrbanSSF [28] and SFANet [11]) perform well in certain categories (e.g., impervious surface, tree and building), their overall performance remains inferior to ours, further validating the signific ant improvements brought by our model design. These gains mainly stem from the cross-level semantic compensation module (CLSCM), which provides global and local semantic compensation, and the decoder-driven auxiliary boundary supervision module (ABSM), which offers auxiliary edge supervision, effectively enhancing segmentation of small targets and complex backgrounds.

**Table 3:** Segmentation results of different models on the Vaihingen dataset. The values in bold represent the top-performing metrics in the table.

Methods	Per-Class IoU(%)					mF1(%)	OA(%)	mIoU(%)
	Imp.surf	Building	Low.veg	Tree	Car			
MANet [51]	92.67	88.25	71.45	81.65	73.67	89.61	92.35	81.54
ABCNet [50]	93.24	91.28	73.07	81.93	78.85	90.60	93.06	83.67
MAResU-Net [30]	94.14	92.07	74.18	82.49	80.16	91.48	93.55	84.61
A2-FPN [29]	93.28	90.26	73.21	81.84	76.48	90.60	92.99	83.15
DC-Swin [54]	94.23	92.47	74.17	82.66	80.27	91.57	93.65	84.76
UNetFormer [10]	94.15	92.11	73.25	81.88	78.80	91.12	93.43	84.04
SFFNet [52]	94.01	92.06	73.37	82.16	81.28	91.46	93.45	84.57
SFANet [11]	94.09	92.78	73.72	82.18	81.99	91.68	93.57	84.95
AFENet [55]	94.01	92.20	74.17	82.79	81.96	91.72	93.63	85.03
BiCoR-Seg [56]	94.15	91.93	74.05	**82.89**	81.77	91.70	93.60	84.96
UrbanSSF [28]	94.20	92.41	74.36	82.65	82.26	91.83	93.78	85.18
BGSC-Net	**94.25**	**92.83**	**74.91**	82.73	**83.25**	**92.08**	**93.80**	**85.61**

**Fig 7:**
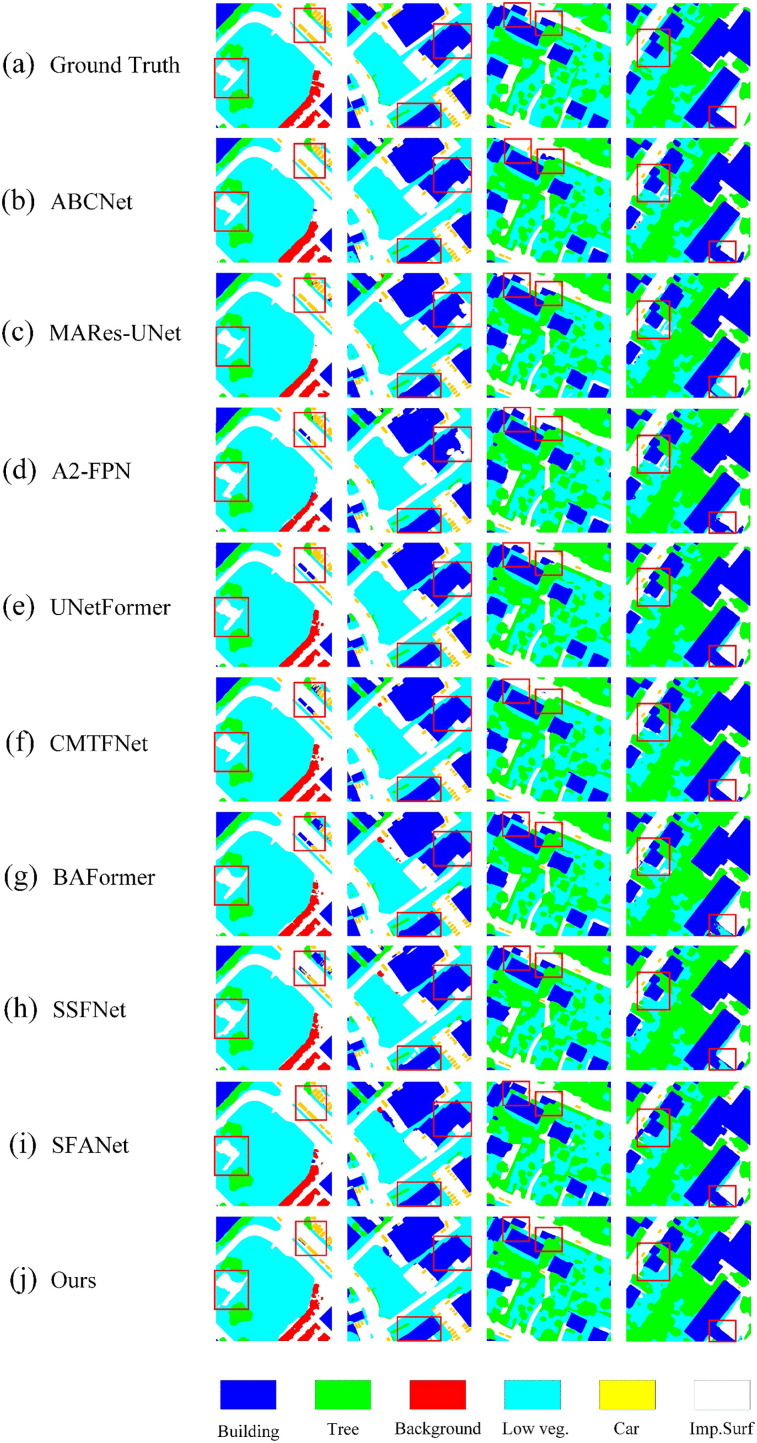
Visualization results of comparative experiments on the Vaihingen dataset.

Fig 7 illustrates the visual segmentation results of BGSC-Net on the Vaihingen dataset, demonstrating the model’s superior performance in boundary delineation and detail preservation. In the first column, under multi-class interwoven scenarios, BGSC-Net achieves clearer and more complete boundaries between impervious surface and low vegetation. Compared to methods such as A2-FPN [27], UNetFormer [10], and DC-Swin [48], it delivers higher segmentation accuracy and reduced class confusion, ensuring smoother transitions between different categories. The model also consistently distinguishes closely adjacent car instances, effectively avoiding confusion between cars and small buildings, which often share similar contours. Furthermore, the remaining columns highlight BGSC-Net’s refined segmentation of building edges. Under complex conditions involving low vegetation and tree interference, the model consistently maintains the structural integrity and continuity of building boundaries. These qualitative results align with the quantitative metrics, confirming the model’s fine-grained segmentation advantage in multi-class scenarios.

Consistent conclusions can be drawn from the quantitative results on the Vaihingen dataset, as reported in Table 4. BGSC-Net achieves the highest Boundary-F1 (71.68%), trimap F-score (76.24%), and Boundary-IoU (46.87%), demonstrating its superior boundary delineation ability across all metrics.The remarkable improvement in Boundary-IoU indicates that BGSC-Net achieves more precise overlap along object contours, which aligns well with the visual results in Fig 7, where clearer and more complete boundaries are observed between impervious surface and low vegetation. Compared with A2-FPN [29], UNetFormer [10], and DC-Swin [54], BGSC-Net significantly reduces class confusion and produces smoother transitions between adjacent categories.In addition, BGSC-Net consistently out-performs UrbanSSF [28], AFENet [55], and BiCoR-Seg [56] on all boundary-related indicators, further validating the effectiveness of the proposed boundary-aware design. The advantage is especially prominent for small objects such as cars. Due to their limited spatial extent and similar contours to nearby buildings, existing methods often produce blurred or broken boundaries. Benefiting from the decoder-driven auxiliary boundary supervision, BGSC-Net generates sharper and more continuous edges, significantly reducing misclassification and boundary fragmentation. This confirms its strong capability in handling densely distributed small targets in complex urban scenes.

**Table 4:** Boundary accuracy comparison of different models on the Vaihingen dataset. The values in bold represent the top-performing metrics in the table.

Methods	Per-Class B-IoU/T-F1(%)					mB-F1(%)	mT-F1(%)	mB-IoU(%)
	Imp.surf	Building	Low.veg	Tree	Car			
MANet [51]	41.65/76.69	40.55/78.33	33.12/67.12	37.14/74.18	56.58/76.30	65.91	74.52	41.80
ABCNet [50]	42.49/76.91	47.99/79.16	34.88/67.31	39.31/74.52	57.28/75.93	68.73	74.76	44.39
MAResU-Net [30]	45.89/78.03	47.27/80.01	34.86/68.01	38.48/74.50	55.30/76.88	69.13	75.48	44.34
A2-FPN [29]	44.90/76.99	46.40/78.94	34.10/67.65	37.05/73.97	56.45/76.55	68.22	74.81	43.78
DC-Swin [54]	46.22/77.89	49.11/79.86	35.60/68.58	39.15/74.63	56.64/76.86	70.14	75.56	45.34
UNetFormer [10]	44.78/77.39	47.59/79.98	34.26/66.97	38.08/74.83	56.12/76.34	68.96	75.10	44.16
SFFNet [52]	44.24/77.64	47.36/79.25	34.18/66.31	37.65/75.11	58.18/76.78	69.07	75.01	44.32
SFANet [11]	44.84/77.44	46.83/75.62	34.64/67.92	**39.44**/74.91	58.26/76.58	69.75	75.29	44.80
AFENet [55]	45.61/77.84	48.78/79.77	34.97/67.24	38.57/75.01	56.97/77.26	70.05	75.42	44.98
BiCoR-Seg [56]	45.81/78.04	49.08/80.07	35.77/68.04	38.87/75.31	57.17/77.46	70.86	75.78	45.76
UrbanSSF [28]	46.73/78.07	49.61/80.21	36.33/**69.56**	39.23/75.29	59.20/76.81	71.39	75.98	46.22
BGSC-Net	**47.09**/77.95	**50.28/80.68**	**36.43**/68.67	39.39/**75.36**	**61.08/78.65**	**71.68**	**76.24**	**46.87**

#### 4.4.3 Experimental results on the Vaihingen dataset.

Table 5 compares the segmentation performance of different models on the LoveDA dataset. BGSC-Net achieves the best overall performance, with a Mean F1 of 70.50%, Overall Accuracy (OA) of 72.08%, and mean IoU (mIoU) of 55.05%, surpassing all mainstream methods. In particular, BGSC-Net achieves notable improvements in the barren and agriculture categories, with IoUs of 38.10% and 60.00%, respectively, demonstrating its high segmentation accuracy under complex background interference. In contrast, although SFANet [11], UrbanSSF [28] and BiCoR-Seg [56] perform relatively well in certain categories (e.g., water or road), their overall scores (Mean F1 and mIoU) remain lower than those of BGSC-Net, further validating the model’s robustness and generalization ability. The strong performance of BGSC-Net is largely attributed to its modular design: the CLSCM integrates multi-scale features through global-local semantic compensation, effectively mitigating class confusion in complex scenes; the ABSM enhances boundary continuity and completeness through edge supervision. The synergy between these modules significantly improves the model’s segmentation performance, particularly for small objects and cluttered environments.

**Table 5:** Segmentation results of different models on the LoveDA dataset. The values in bold represent the top-performing metrics in the table.

Methods	Per-Class IoU(%)							mF1(%)	OA(%)	mIoU(%)
	Background	Building	Road	Water	Barren	Forest	Agriculture			
MANet [51]	52.35	59.29	52.45	68.01	26.68	41.49	48.21	65.54	68.30	49.78
ABCNet [50]	53.22	61.55	51.81	62.09	27.53	41.85	48.81	65.47	68.21	49.55
A2-FPN [29]	52.24	53.76	53.83	69.70	33.45	43.69	50.54	67.42	69.00	51.58
UNetFormer [10]	53.14	59.24	53.07	66.63	27.78	43.63	50.73	66.38	68.92	50.60
DecoupleNet [53]	53.85	62.29	53.34	66.05	24.14	42.66	50.38	65.94	69.17	50.39
BAFormer [24]	53.84	63.31	54.34	69.14	34.71	44.42	52.58	68.81	70.43	53.19
SFFNet [52]	52.22	61.95	55.95	64.17	28.22	43.44	47.74	66.32	67.94	50.53
SFANet [11]	53.79	63.65	**57.00**	65.31	33.25	41.63	57.64	68.57	71.19	53.18
AFENet [55]	53.72	63.02	56.54	68.78	36.56	40.05	55.13	68.93	70.57	53.40
BiCoR-Seg [56]	53.71	63.25	56.58	69.12	37.28	41.45	57.32	69.78	71.15	54.12
UrbanSSF [28]	53.87	63.43	56.55	**69.73**	36.87	42.02	58.24	70.03	71.62	54.39
BGSC-Net	**53.89**	**63.77**	56.61	69.67	**38.10**	**43.29**	**60.00**	**70.50**	**72.08**	**55.05**

**Fig 8:**
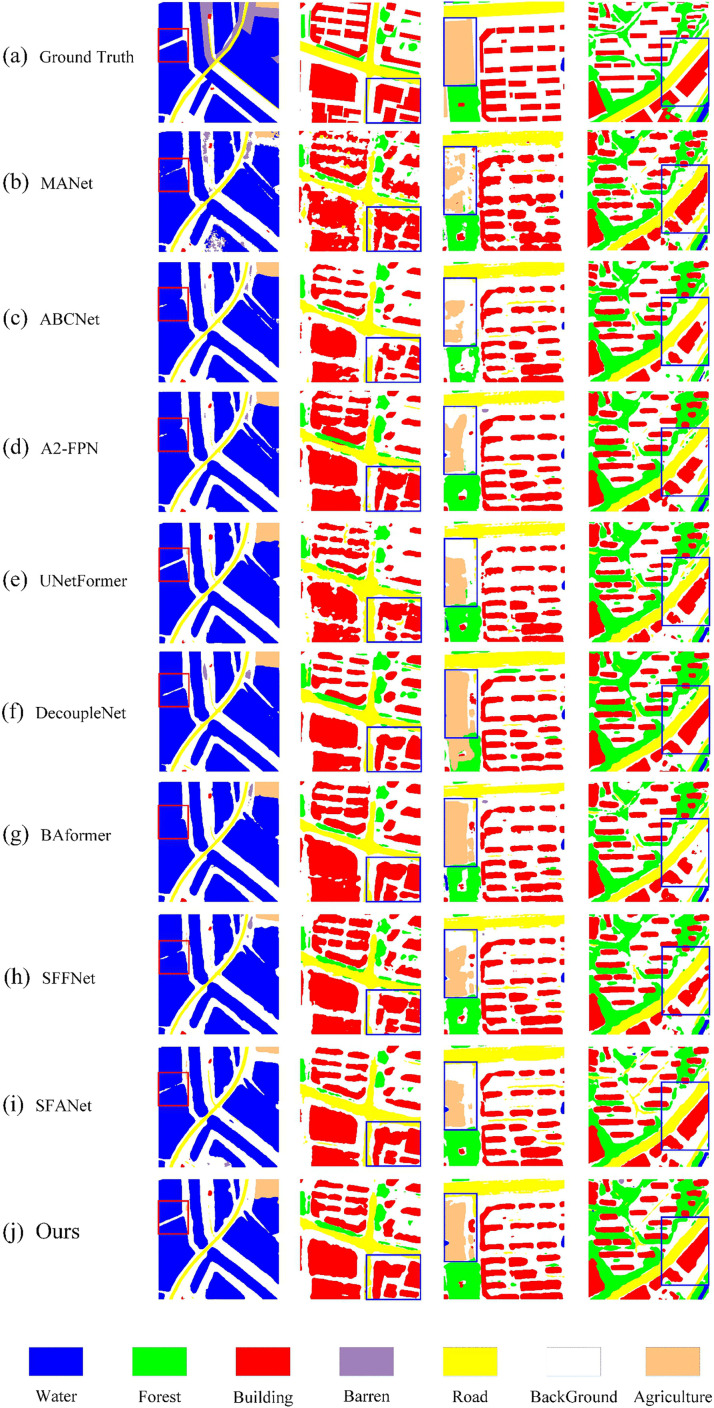
Visualization results of comparative experiments on the LoveDA dataset.

Fig 8 presents the visual segmentation results of BGSC-Net on the LoveDA dataset, providing an intuitive validation of its performance in complex scenes involving multiple categories. In the first column, BGSC-Net generates clearer and more complete boundaries for the water category, and accurately segments narrow background areas such as rural roads, effectively addressing the common issue of incomplete small-object segmentation seen in other methods. The second and fourth columns highlight the model’s precise delineation of building boundaries under interference from forest and agriculture, maintaining the continuity of building edges. The third column demonstrates the model’s excellent segmentation performance in regions where barren and agriculture are intertwined, effectively avoiding class confusion and boundary fragmentation. These results align with the quantitative metrics, comprehensively demonstrating BGSC-Net’s superior capability in multi-category segmentation under complex backgrounds.

#### 4.4.4. Experimental results on the UAVid dataset.

Table 6 presents the segmentation results on the UAVid dataset. BGSC-Net achieves the highest performance, with a MeanF1 of 85.06%, OA of 89.41%, and mIoU of 74.77%, significantly outperforming other models. It excels in key traffic-related categories,such as Road (81.53%), Moving Car (77.80%), and Human (50.30%), demonstrating strong robustness against occlusion and multi-object interference in complex urban scenes. Compared to MANet [51], DecoupleNet [53], and A2-FPN [29], BGSC-Net shows clear advantages in detecting small and occluded objects. It also surpasses DC-Swin [54] and SFANet [11] in static categories like building and static car, reflecting more balanced and generalized performance across classes. Although SFFNet [52] and DC-Swin [54] perform relatively well in certain categories such as building, vegetation, or human, their overall scores in terms of MeanF1 and mIoU remain lower than those of BGSC-Net.These improvements are attributed to BGSC-Net’s modular design, which enhances its adaptability to diverse targets, varying scales, and complex backgrounds, confirming its strong competitiveness on the UAVid dataset. Furthermore, BGSC-Net consistently outperforms recently proposed methods, including the state-space model based UrbanSSF and the newly proposed AFENet and BiCoR-Seg. In particular, BGSC-Net shows more stable performance in traffic-related and small-object categories, indicating stronger robustness to motion blur and occlusion. These results demonstrate that although UrbanSSF benefits from long-range dependency modeling, and AFENet and BiCoR-Seg introduce advanced feature enhancement strategies, BGSC-Net achieves better overall performance due to its effective multi-scale feature fusion and boundary-aware supervision.

**Table 6:** Segmentation results of different models on the UAVid dataset. The values in bold represent the top-performing metrics in the table.

Methods	Per-Class IoU(%)								mF1(%)	OA(%)	mIoU(%)
	Clutter	Building	Road	Tree	Vegetation	Moving Car	Static Car	Human			
MANet [51]	59.94	89.93	74.16	77.18	66.82	70.26	67.86	48.09	81.30	86.27	69.28
A2-FPN [29]	65.51	91.36	78.14	78.78	69.02	71.33	69.00	48.16	82.76	88.00	71.41
DC-Swin [54]	68.34	**93.28**	80.22	79.97	**72.04**	75.87	71.67	47.66	84.21	89.39	73.63
UNetFormer [10]	64.94	91.60	77.91	78.69	69.24	73.17	69.65	48.02	82.92	87.96	71.65
DecoupleNet [53]	62.91	90.58	78.37	77.98	69.85	71.47	68.74	46.82	82.33	87.49	70.84
CGGLNet [57]	65.35	91.62	79.01	78.84	70.48	74.30	71.65	47.98	83.42	88.21	72.58
SFFNet [52]	67.18	92.02	80.45	79.42	69.72	76.47	72.95	49.13	84.05	88.69	73.32
SFANet [11]	66.89	92.59	80.23	79.27	70.78	77.11	74.51	48.03	84.25	88.80	73.68
AFENet [55]	67.35	92.36	80.49	78.59	69.24	73.75	71.65	49.76	83.95	88.54	72.90
BiCoR-Seg [56]	67.82	92.71	81.12	79.35	70.18	75.63	73.45	50.08	84.35	88.97	73.78
UrbanSSF [28]	67.64	92.82	80.97	79.08	70.87	76.11	73.80	50.10	84.53	88.86	73.98
BGSC-Net	**68.50**	93.03	**81.53**	**79.97**	71.45	**77.80**	**74.96**	**50.30**	**85.06**	**89.41**	**74.77**

**Fig 9:**
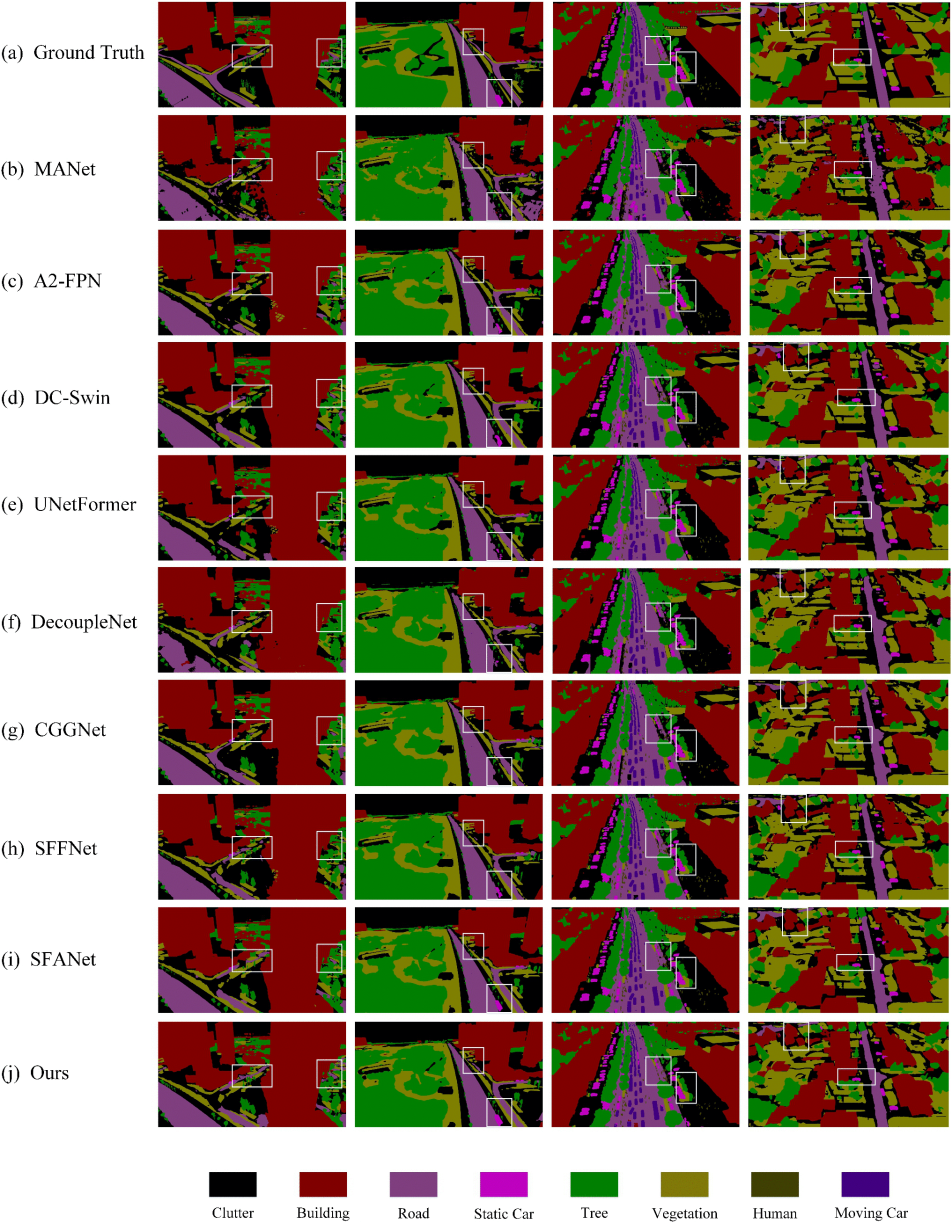
Visualization results of comparative experiments on the UAVid dataset.

Fig 9 shows the visual segmentation results of BGSC-Net on the UAVid dataset, highlighting its robustness in complex traffic scenes. In the first column, BGSC-Net accurately delineates continuous road regions despite occlusions from buildings, billboards, and trees, outperforming other models that suffer from fragmentation and misclassification. The second and fourth columns demonstrate its effectiveness in multi-class overlapping areas, particularly in preserving building boundaries, reflecting the contribution of the ABSM to edge perception and supervision. In the third column, BGSC-Net distinguishes visually similar classes like tree and vegetation, even under occlusion near bus stops, accurately detecting small human targets and reducing class confusion. These improvements stem from the CLSCM, which enhances global-local semantic interaction and strengthens the model’s ability to handle small objects and complex backgrounds.Overall, BGSC-Net exhibits strong and generalizable segmentation performance under multi-class, cluttered, and occluded conditions, validating the effectiveness of its modular design.

#### 4.4.5. Experimental results on the MSFSD.

Table 7 presents the semantic segmentation results on the Mangrove Species Fine-grained Segmentation Dataset (MSFSD). The proposed BGSC-Net achieves the highest overall performance, with a mean F1-score (mF1) of 94.42%, overall accuracy (OA) of 94.25%, and mean Intersection over Union (mIoU) of 89.58%, significantly outperforming all comparative methods.At the species level, BGSC-Net attains the best IoU across four of the five mangrove species, including Sonneratia apetala (89.94%), Avicennia marina (87.87%), Kandelia obovata (83.37%), and Bruguiera gymnorhiza (92.36%), and performs comparably in Rhizophora stylosa (93.94%). These results demonstrate the model’s strong capability in distinguishing morphologically similar mangrove species, especially for Avicennia marina, where it surpasses the second-best SFANet [11] by 9.94% in IoU. While SFANet [11] shows competitive results in Sonneratia apetala and Rhizophora stylosa, its overall mIoU (86.86%) remains lower than that of BGSC-Net.The performance gain can be attributed to the effective collaboration of the CLSCM and ABSM modules, which enhance feature representation for fine-grained

**Table 7:** Segmentation results of different models on the MSFSD. The values in bold represent the top-performing metrics in the table.

Methods	Per-Class IoU(%)					mF1(%)	OA(%)	mIoU(%)
	K. obovata	S. apetala	A. marina	B. gymnorhiza	R. stylosa			
MANet [51]	80.23	84.89	73.42	91.54	92.76	91.47	92.91	84.57
ABCNet [50]	81.61	85.21	73.00	90.05	92.73	91.45	92.42	84.52
UNetFormer [10]	81.73	84.49	77.14	91.55	93.56	92.18	93.27	85.69
CGGLNet [57]	81.91	85.43	72.77	90.93	94.11	91.73	93.17	85.03
SFFNet [52]	82.91	85.83	74.59	91.65	93.95	92.20	93.50	85.79
SFANet [11]	82.00	88.01	77.93	91.87	**94.49**	92.85	93.51	86.86
AFENet [55]	78.87	83.80	73.64	91.94	92.93	91.18	92.71	84.08
BiCoR-Seg [56]	83.16	87.29	77.36	91.84	93.83	92.72	93.88	86.64
UrbanSSF [28]	83.06	89.34	81.27	91.95	94.18	93.51	94.05	87.96
BGSC-Net	**83.37**	**89.94**	**87.87**	**92.36**	93.94	**94.42**	**94.25**	**89.58**

species with high inter-class similarity and improve boundary delineation in dense canopy regions. The results confirm that BGSC-Net exhibits strong competitiveness and ecological applicability in the challenging task of mangrove species segmentation.

**Fig 10:**
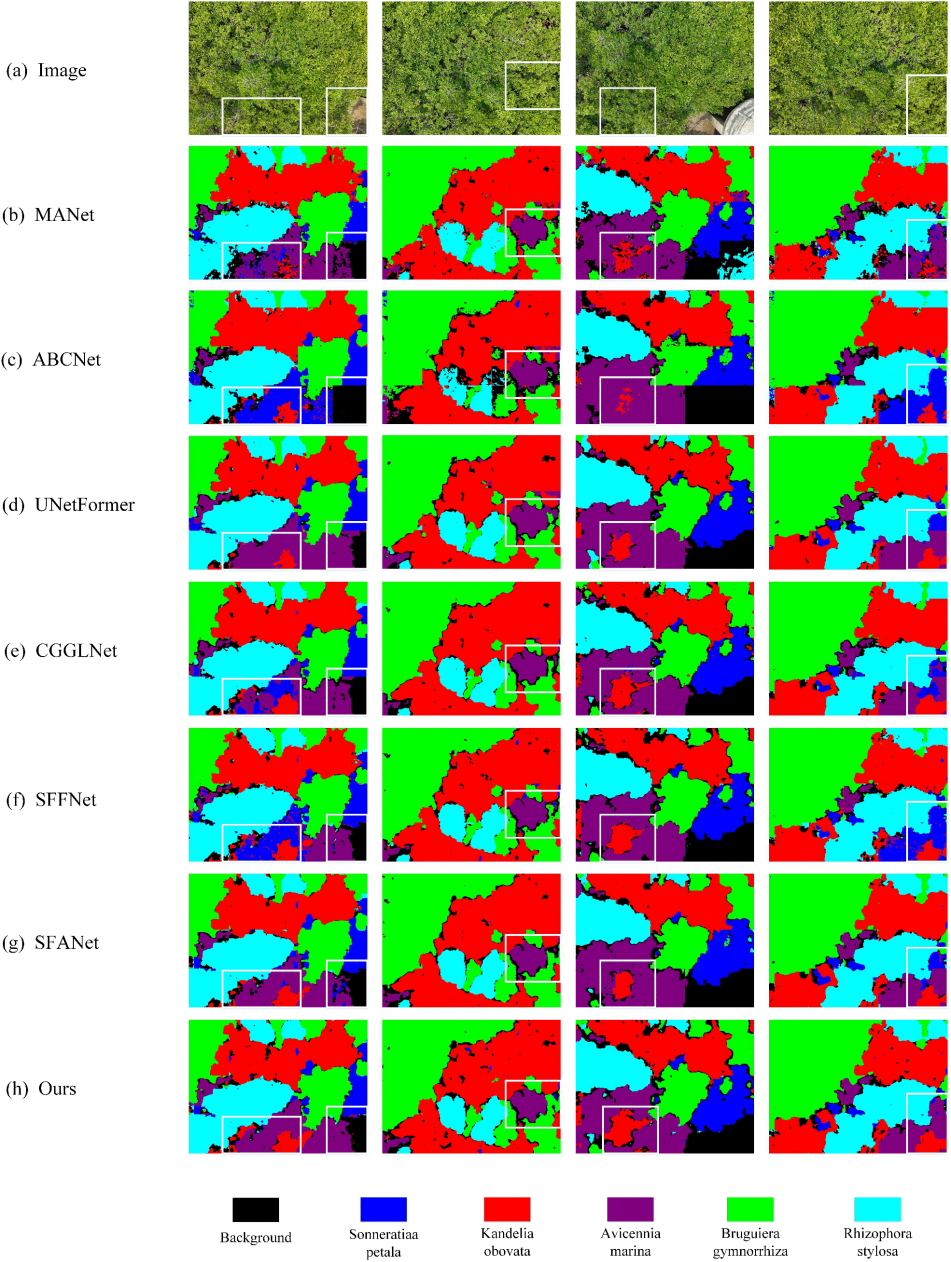
Visualization results of comparative experiments on the MSFSD.

Fig 10 presents the visual segmentation results of BGSC-Net on the Mangrove Species Fine-grained Segmentation Dataset (MSFSD), demonstrating its discriminative capability in complex mangrove species identification. In the first and second rows, BGSC-Net accurately distinguishes Sonneratia apetala and Avicennia marina in densely mixed canopies, significantly reducing inter-species confusion present in other methods. The clear segmentation of Kandelia obovata crowns in the third row highlights the model’s sensitivity to fine-grained morphological differences, where comparative models exhibit varying degrees of misclassification or boundary blurring. Furthermore, BGSC-Net maintains precise boundary delineation between Bruguiera gymnorrhiza and Rhizophora stylosa in overlapping regions, as shown in the fourth and fifth rows, underscoring the contribution of the ABSM module to edge integrity. These visual improvements confirm the effectiveness of the CLSCM in enhancing feature representation for species with high inter-class similarity, while the ABSM ensures structural continuity in complex canopy environments. Overall, BGSC-Net achieves ecologically meaningful segmentation in species-level mangrove mapping, validating its practical value for fine-grained ecological monitoring.

### 4.5. Ablation experiments

To verify the effectiveness of BGSC-Net, ablation studies were performed on five datasets: Potsdam, Vaihingen, LoveDA, UAVid and the self-constructed MSFSD. We assessed the individual and combined contributions of the cross-level semantic compensation module (CLSCM)) and decoder-driven auxiliary boundary supervision module (ABSM). As shown in Tables 8–12 (the values in bold represent the top-performing metrics in the table), key metrics such as mF1 and mIoU steadily improved with the addition of each module, confirming their positive impact on overall segmentation performance.

**Table 8:** Ablation experiments on the Potsdam dataset.

Methods	Component		MeanF1(%)	OA(%)	mIoU(%)
	CLSCM	ABSM			
Potsdam			92.59	91.30	86.44
	√		93.07	91.91	87.27
		√	92.99	91.94	87.11
	√	√	93.24	91.95	87.57

**Table 9:** Ablation experiments on the Vaihingen dataset.

Methods	Component		MeanF1(%)	OA(%)	mIoU(%)
	CLSCM	ABSM			
Vaihingen			91.33	93.51	84.37
	√		91.90	93.72	85.29
		√	91.80	93.60	85.15
	√	√	92.00	93.75	85.47

**Table 10:** Ablation experiments on the LoveDA dataset.

Methods	Component		MeanF1(%)	OA(%)	mIoU(%)
	CLSCM	ABSM			
LoveDA			69.00	70.47	53.65
	√		69.53	71.85	54.33
		√	69.99	71.78	54.71
	√	√	70.50	72.08	55.05

**Table 11:** Ablation experiments on the UAVid dataset.

Methods	Component		MeanF1(%)	OA(%)	mIoU(%)
CLSCM	ABSM
UAVid			83.63	88.91	72.70
	√		84.64	88.92	74.15
		√	84.80	89.21	74.39
	√	√	84.94	89.31	74.63

**Table 12:** Ablation experiments on the MSFSD.

Methods	Component		MeanF1(%)	OA(%)	mIoU(%)
	CLSCM	ABSM			
MSFSD			92.98	93.61	87.22
	√		93.58	93.91	88.36
		√	93.78	94.01	88.48
	√	√	94.42	94.25	89.58

Effectiveness of CLSCM: Integrating CLSCM into the EfficientNet encoder enables efficient fusion of semantic information across layers, effectively addressing semantic misalignment and improving small-object detection. This leads to enhanced recognition of fine-grained structures. Quantitatively, CLSCM contributes consistent performance gains: on the Potsdam dataset, mF1 increases by 0.48% and mIoU by 0.83%; on Vaihingen, mF1 improves by 0.57% and mIoU by 0.92%; on LoveDA, mF1 rises by 0.53% and mIoU by 0.68%; and on UAVid, mF1 increases by 1.01% and mIoU by 1.45%. Notably, on the MSFSD, the introduction of CLSCM boosts mF1 by 0.60% and mIoU by 1.14%, significantly improving the discrimination of morphologically similar species such as Avicennia marina and Sonneratia apetala. These results validate the effectiveness of the semantic compensation mechanism in enhancing segmentation performance.

Effectiveness of ABSM: Integrating the auxiliary boundary supervision module (ABSM) into the Transformer decoder enhances boundary representation through edge-aware feature refinement and auxiliary supervision. This design effectively improves boundary continuity, integrity, and class separability, particularly in complex and cluttered remote sensing scenes. Quantitatively, ABSM yields consistent performance improvements: on the Potsdam dataset, mF1 increases by 0.40% and mIoU by 0.67%; on Vaihingen, mF1 improves by 0.47% and mIoU by 0.78%; on LoveDA, mF1 rises by 0.99% and mIoU by 1.06%; and on UAVid, mF1 increases by 1.17% and mIoU by 0.69%. On the MSFSD, ABSM elevates mF1 by 0.80% and mIoU by 1.26%, markedly enhancing the boundary delineation accuracy of intertwined mangrove canopies. These results validate the effectiveness of ABSM in enhancing boundary segmentation under challenging conditions. To further validate the contribution of key components within ABSM, we conducted systematic ablation studies on the Vaihingen dataset.The experiments focus on three core design aspects: multi-stage bridging strategies (integration of decoder features from different stages), upsampling and smoothing operations (pixelshuffle and average pooling), and the edge-aware feature enhancement mechanism (EFEM). Results are presented in Table 13 and Table 14. Table 13 demonstrates the importance of hierarchical feature integration.The experimental results indicate that the complete three-stage integration ($D_{\text{1}}$, $D_{\text{2}}$, and $D_{\text{3}}$) achieves the highest scores across all metrics, confirming that multi-scale context fusion is essential for robust boundary representation in complex scenes. Table 14 examines the contributions of upsampling/ smoothing techniques and the EFEM module. The baseline with simple convolution performs poorly in boundary metrics. Introducing pixelshuffle upsampling alone improves mB-IoU by 0.65%, demonstrating its effectiveness in enhancing semantic continuity through channel-to-spatial redistribution. Average pooling smoothing alone provides moderate gains by suppressing prediction noise. Their combination yields further improvement, indicating complementary roles in boundary refinement. Most notably, integrating the complete EFEM module brings the most substantial performance leap, boosting mB-IoU by 1.49% over the Pixelshuffle+AvgPool configuration. This validates EFEM’s critical role in explicit edge modeling through attention-based feature enhancement.These findings confirm that ABSM’s effectiveness stems from the synergistic combination of hierarchical feature fusion, advanced upsampling techniques, and explicit edge-aware enhancement.

**Table 13:** Ablation study on multi-stage bridging strategies in ABSM.

Methods	Input			mB-F1(%)	mT-F1(%)	mB-IoU(%)
	D1	D2	D3			
**Vaihingen**	√			69.98	75.52	45.34
		√		70.11	75.76	45.51
			√	69.95	75.35	45.23
	√	√		70.30	75.79	45.62
	√		√	70.14	75.68	45.33
		√	√	70.42	75.80	45.70
	√	√	√	**71.68**	**76.24**	**46.87**

**Table 14:** Ablation study on the key components of ABSM.

Methods	Component			mB-F1(%)	mT-F1(%)	mB-IoU(%)
	Pixelshuffle	AvgPool	EFEM			
**Vaihingen**				69.63	74.12	44.80
	√			70.20	75.70	45.45
		√		69.87	75.32	45.16
			√	70.12	75.73	45.29
	√	√		70.46	75.81	45.78
	√		√	70.57	75.98	45.96
		√	√	70.43	75.67	45.75
	√	√	√	**71.68**	**76.24**	**46.87**

Synergistic Effect of Module Integration: Combining CLSCM and ABSM leads to further performance gains. On the Potsdam dataset, mF1 and mIoU increased by 0.65% and 1.13%, respectively; on Vaihingen, by 0.67% and 1.10%; on LoveDA, by 1.50% and 1.40%; and on UAVid, by 1.31% and 1.93%. On the MSFSD, the full model achieves optimal performance, with mF1 and mIoU reaching 94.42% and 89.58%, respectively, representing a significant improvement over the baseline model. These results confirm the complementary strengths of the two modules and their overall benefit to segmentation performance.

**Fig 11:**
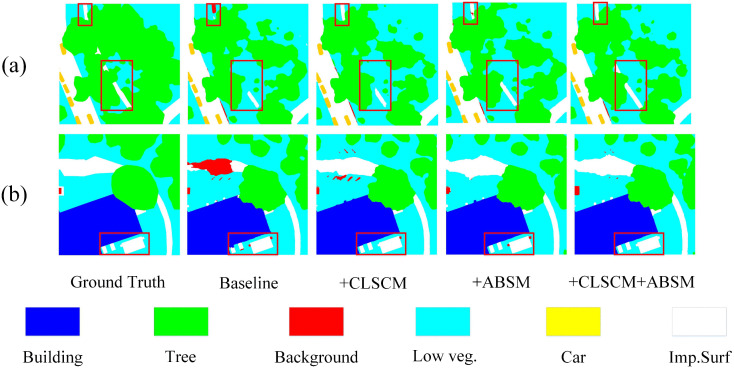
Visualization results of ablation experiments on the Potsdam dataset.

**Fig 12:**
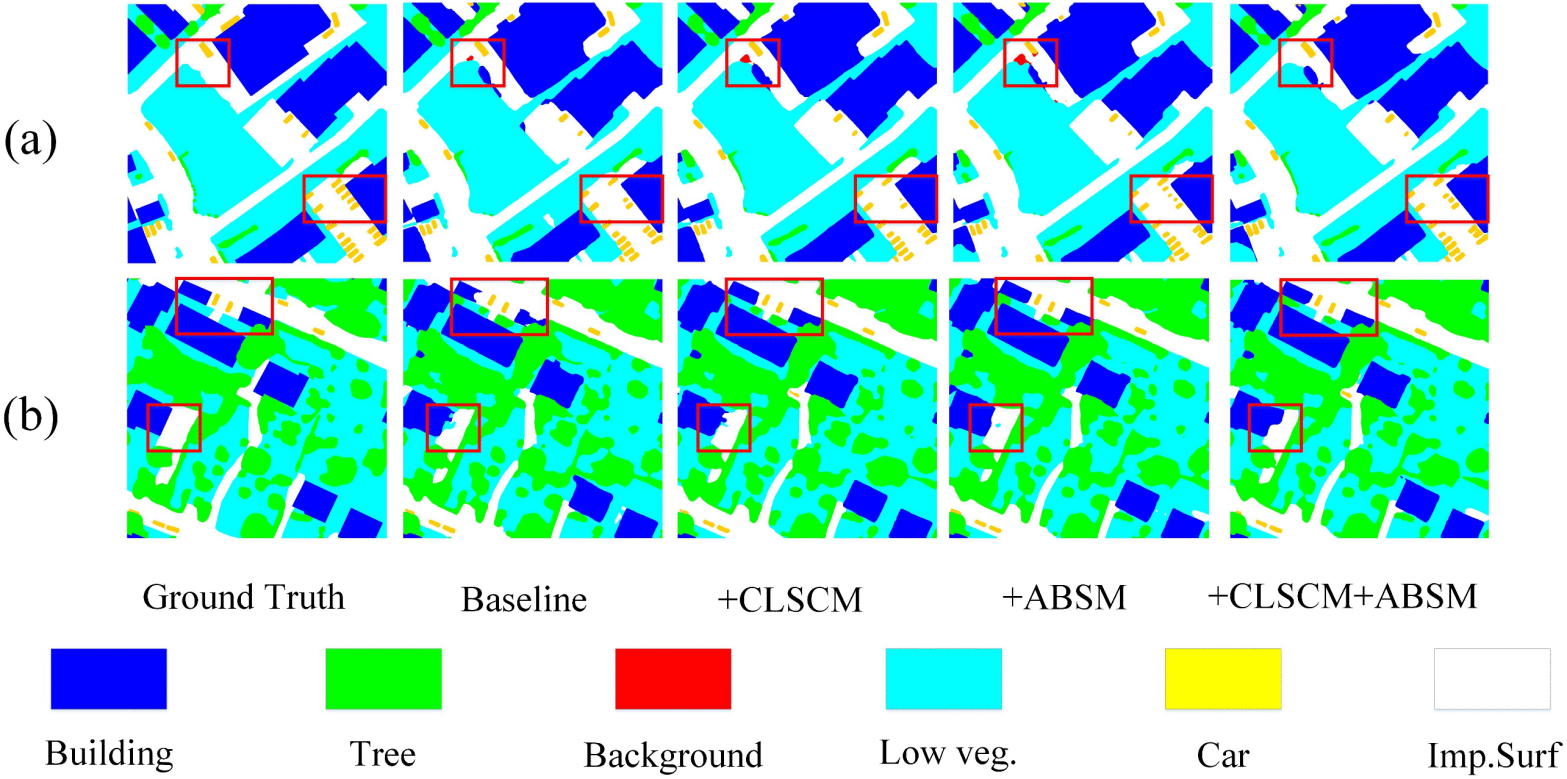
Visualization results of ablation experiments on the Vaihingen dataset.

**Fig 13:**
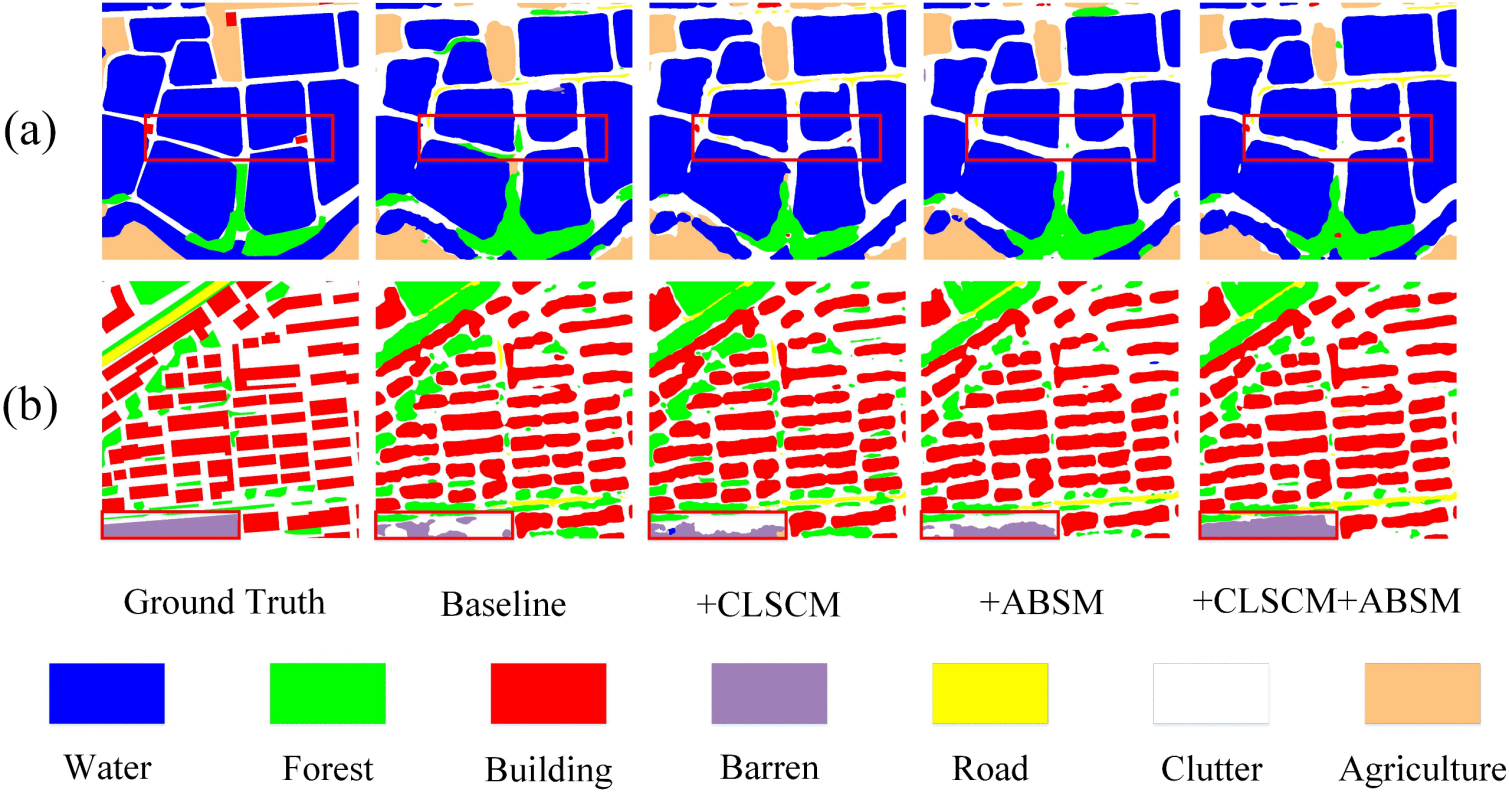
Visualization results of ablation experiments on the LoveDA dataset.

**Fig 14:**
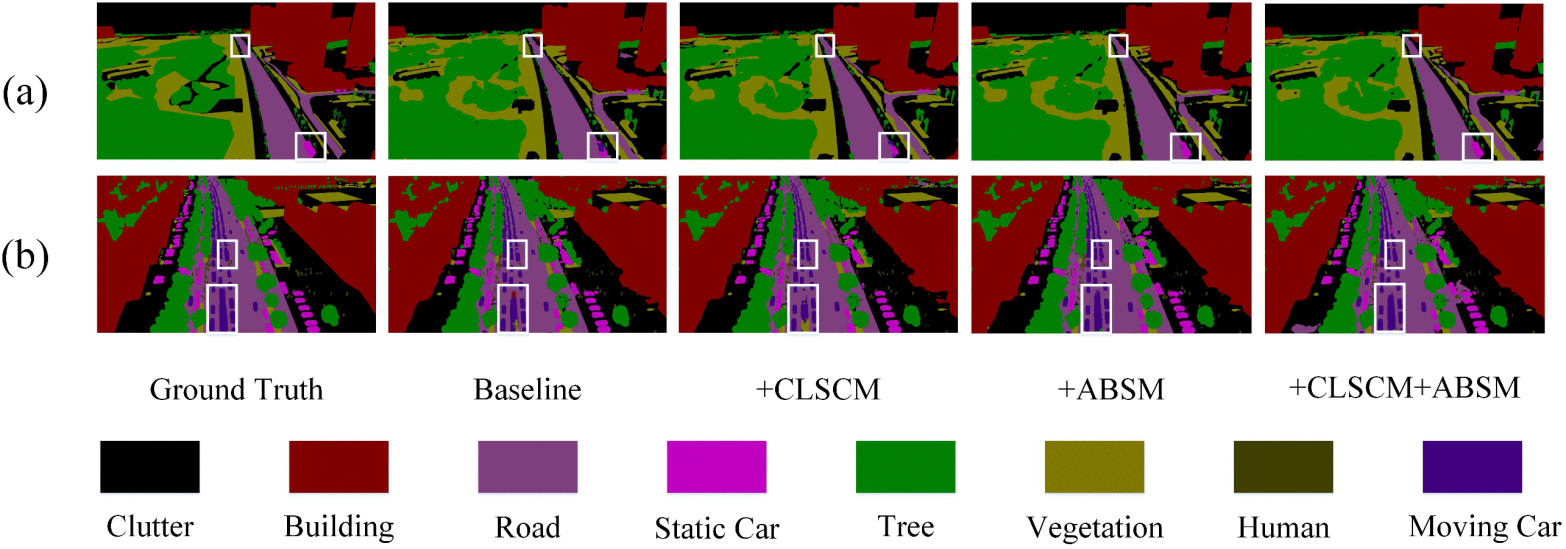
Visualization results of ablation experiments on the UAVid dataset.

**Fig 15:**
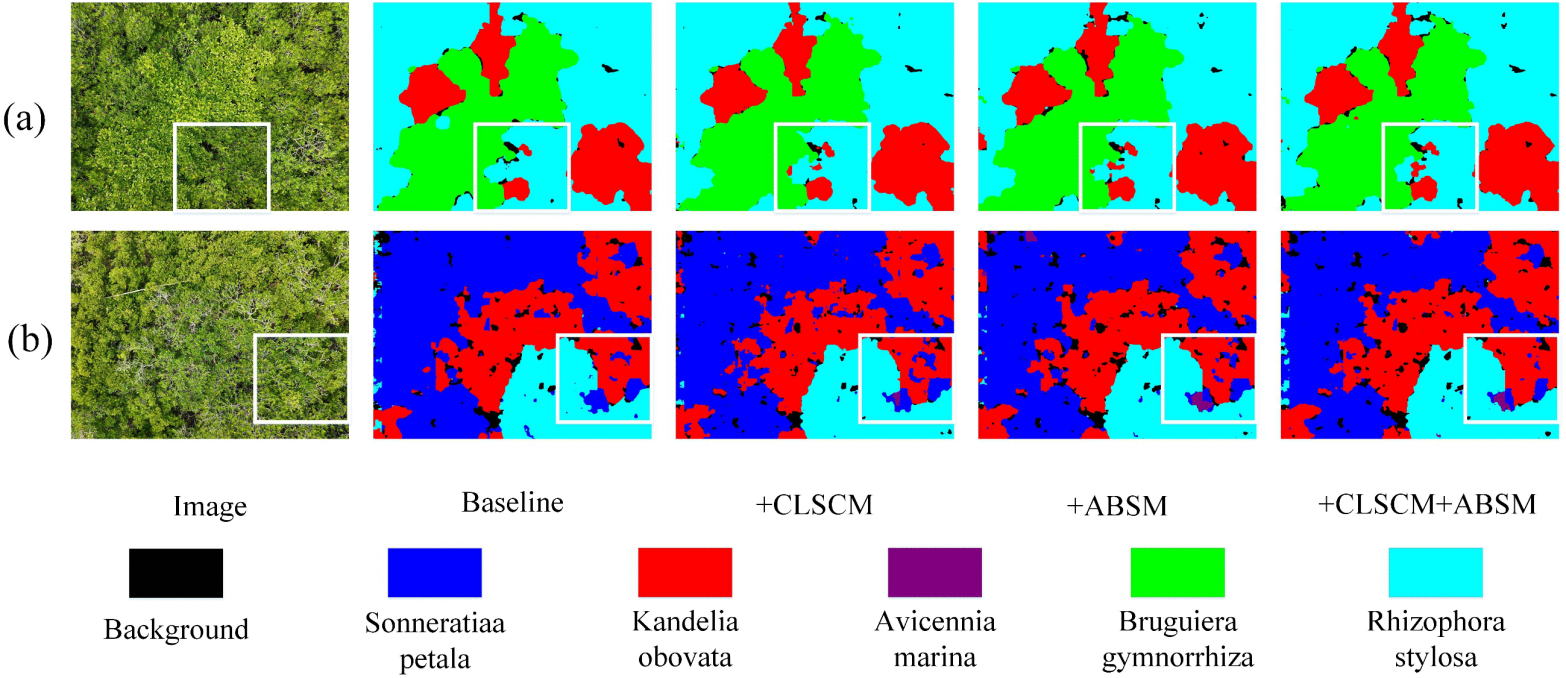
Visualization results of ablation experiments on the MSFSD.

Visual Result Analysis: Fig 11 to 15 systematically illustrate the performance improvements brought by each module from a visual perspective. The results show that as modules are progressively added, small-object segmentation becomes more detailed, object boundaries more complete, and inter-class confusion significantly reduced. Specifically, Fig 11(b) demonstrates that the CLSCM effectively suppresses false detections in small-object categories like background and impervious surface. Similarly, the highlighted regions in Fig 12, 13, and 14(a) validate CLSCM’s improvements in segmenting small buildings and recognizing multiple parallel vehicles under shadowed conditions. In Fig 13(b), the model correctly distinguishes vegetables on a truck-misclassified as vegeta-tion by other methods-highlighting its superiority in handling complex small-object seg-mentation and showcasing the benefits of semantic compensation. Fig 11(a) further reveals CLSCM’s strength in pedestrian segmentation within complex traffic scenes. On the MSFSD, as shown in Fig 15(a), the baseline model exhibits significant confusion between species such as Sonneratia apetala and Avicennia marina. The incorporation of CLSCM significantly enhances the recognition of species with fine-leaf structures, such as Avicennia marina and Kandelia obovata, reducing misclassification between species.The ABSM also contributes noticeably through auxiliary boundary supervision. It significantly enhances boundary integrity and continuity, thereby improving segmentation accuracy. For example, in Fig 11(a), road boundaries appear more continuous and intact under occlusion by trees and low vegetation. In Fig 12(b), ABSM alleviates the edge blurring and fragmentation of Barren areas, visually confirming its positive effect on boundary refinement. In the context of mangrove species segmentation in Fig 15(a), ABSM further refines the canopy boundaries between Bruguiera gymnorrhiza and Rhizophora stylosa, resulting in more complete and clearer segmentation contours. Ultimately, the full model achieves the best segmentation performance across all species, particularly in distinguishing morphologically similar species. In summary, CLSCM and ABSM tackle multi-scale semantic misalignment, boundary ambiguity, and class confusion through global-local semantic compensation and boundary refinement. Their synergistic design not only enhances the overall segmentation performance of BGSC-Net but also demonstrates the practicality and superiority of modular design in remote sensing image segmentation tasks.

### 4.6. Model complexity analysis

To assess BGSC-Net’s efficiency and accuracy, we evaluate its FLOPs and parameter count. Table 15 compares multiple models on the LoveDA dataset using identical inputs (two 512 × 512 patches) to ensure fair benchmarking. BGSC-Net records 8.40 GFLOPs and 10.86 M parameters, slightly higher than lightweight models like SFANet (7.04 GFLOPs) and DecoupleNet (6.73 M), but far more efficient than heavy models like BAFormer (148.62 GFLOPs) and MANet (77.76 GFLOPs), indicating strong deployability. In terms of accuracy, BGSC-Net achieves the best performance, with a MeanF1 of 70.50% and mIoU of 55.05%, outperforming BAFormer (+1.69% MeanF1, + 1.86% mIoU) and SFANet (+1.93%, + 1.87%). Overall, BGSC-Net offers an excellent trade-off between performance and complexity, making it ideal for precision-demanding remote sensing tasks in resource-constrained settings.

**Table 15:** Computational complexity analysis on a single NVIDIA GeForce RTX4090 GPU. The values in bold represent the top-performing metrics in the table.

Methods	FLOPs(G)	Params(M)	MeanF1(%)	mIoU(%)
MANet	77.76	35.86	65.54	49.78
ABCNet	15.63	13.39	65.47	49.55
A2-FPN	41.83	22.82	67.42	51.58
UNetFormer	11.74	11.68	66.38	50.60
DecoupleNet	8.03	6.73	65.94	50.39
BAFormer	148.62	35.08	68.81	53.19
SFFNet	15.10	14.43	66.32	50.53
SFANet	7.04	10.57	68.57	53.18
BGSC-Net	8.40	10.86	70.50	55.05

## 5. Conclusions

This paper proposed an efficient boundary-guided semantic compensation network (BGSC-Net) to address two key challenges in high-resolution remote sensing imagery: the frequent omission of small targets and blurred category boundaries. By introducing a cross-level semantic compensation module (CLSCM) and a decoder-driven auxiliary boundary supervision module (ABSM), which effectively bridges the gap between low-level spatial details and high-level semantics, enhancing its perception of complex structures and boundary regions. The experimental results on multiple remote sensing datasets have demonstrated that BGSC-Net significantly outperforms existing methods in terms of MeanF1 and mIoU, while maintaining low computational complexity (8.40 GFLOPs) and a compact parameter size (10.86M), highlighting its strong balance between accuracy and efficiency for practical deployment. It is particularly noteworthy that experiments on the self-constructed Mangrove Species Fine-grained Segmentation Dataset (MSFSD) further validate the strong generalization capability of BGSC-Net. The model demonstrates outstanding performance in distinguishing morphologically similar mangrove species, achieving significant improvements in the recognition of easily confused species such as Avicennia marina and Aegiceras corniculatum, confirming the effectiveness of its semantic compensation and boundary enhancement mechanisms in ecological fine-grained identification tasks.

Nonetheless, BGSC-Net still has several aspects worth further exploration. Its seg-mentation performance can fluctuate in densely populated and category-rich urban–rural areas, suggesting limited feature discrimination under multi-class coexistence. Additionally, in scenes with large scale variation, such as the coexistence of large buildings and tiny facilities, the fixed receptive field limits the model’s representational capacity. In mangrove species segmentation, the model’s adaptability to partially submerged canopies under tidal conditions and morphological changes at different growth stages still requires enhancement. Furthermore, its generalization to more complex urban–rural mixed scenes remain to be validated.

Future research can be carried out in the following directions: First, introducing target density-aware mechanisms and dynamic receptive field adjustment strategies may further enhance the model’s adaptability to dense targets and multi-scale structures. Second, integrating multi-modal remote sensing data can improve the model’s ability to represent complex land cover semantics and spatial structures. Third, exploring more efficient lightweight designs, such as structural pruning, sparse attention, and knowledge distillation, can help reduce deployment costs on edge devices. Particularly for mangrove species identification, future work could combine hyperspectral and LiDAR data to establish a multi-modal species recognition benchmark and investigate the incorporation of species distribution priors and ecological knowledge to enhance recognition robustness in complex intertidal environments.

In summary, BGSC-Net has demonstrated strong performance in both accuracy and efficiency for remote sensing semantic segmentation tasks. It provides new insights into addressing key challenges such as small-object recognition, boundary ambiguity, and multi-scale feature fusion in remote sensing imagery. With the future integration of real-world constraints and cross-modal information, BGSC-Net holds promising application potential in diverse remote sensing tasks, including fine-grained urban planning and rural resource monitoring. With the further integration of real-world constraints and cross-modal information, the network holds promising application potential in diverse remote sensing tasks, including fine-grained urban planning, rural resource monitoring, and dynamic monitoring of mangrove ecosystems.
